# Obstacles and facilitators of return to work among people with persistent pain who receive benefit payments: an in-depth interview study

**DOI:** 10.1186/s12889-025-24747-0

**Published:** 2025-10-21

**Authors:** Pål André Amundsen, Pernille Marie Stähr Irgens, Kim Burton, Ira Malmberg-Heimonen, Robert Froud

**Affiliations:** 1 School of Health Sciences, Kristiana University College, Oslo, Norway; 2 Professor of Occupational Healthcare, University of Huddersfield, Huddersfield, UK; 3 Department of Social Work, Child Welfare and Social Policy, Oslo Metropolitan University, Oslo, Norway; 4 Warwick Clinical Trials Unit, Warwick Medical School, University of Warwick, Coventry, UK

**Keywords:** Chronic pain, Persistent pain, Return to work, Qualitative research, Semi-structured interviews, Health service research

## Abstract

**Background:**

Persistent pain is a major cause of work disability and early retirement, posing considerable challenges to welfare systems. The process of returning to work is complex and multifaceted, often becoming more difficult as the duration of absence increases. Most research on facilitators and obstacles for returning to work has focused on individuals on sick leave; less is known about those who are unemployed and receiving benefit payments. The aim in this study was to explore the obstacles and facilitators faced by participants with persistent pain, who are attempting to return to work while receiving benefit payments.

**Methods:**

In this descriptive qualitative study, we used purposive sampling from a cohort study on the impact of being unemployed due to persistent pain. Semi-structured interviews were conducted using a topic guide, audio-recorded, transcribed verbatim and analysed using the Framework method.

**Results:**

We interviewed 29 participants, of whom 12 had partly or fully returned to work, while 17 had not. Themes emerged around participants’ experiences of obstacles and facilitators: (1) Norwegian Labour and Welfare Administration, (2) healthcare, (3) psychological, (4) pain, and (5) perceptions of work. For facilitators, additional themes of ‘luck’ and ‘disability pension’ emerged.

**Conclusion:**

While struggling with pain and coexisting health issues, participants experienced overwhelming system and contextual obstacles. Key facilitators included fortuitous encounters with supportive welfare system staff and personal attributes such as psychological flexibility and determination. Our findings highlight areas of the welfare system that could be improved to provide more empathetic and person-centred pathways for people with persistent pain wanting to return to work.

**Supplementary Information:**

The online version contains supplementary material available at 10.1186/s12889-025-24747-0.

## Background

Persistent pain, often musculoskeletal, represents a challenge for individuals and society, with substantial healthcare and social costs [1–3]. It is a significant cause of work disability and early retirement and challenges welfare systems [4, 5]. Having a job is important for health, identity, social role, and status [6]. Being out of work increases risk of poor physical and mental health [6–8]. Return to work (RTW) can aid recovery, improve quality of life, and reduce poverty [6, 9]. However, RTW is a complex multifaceted process; the probability of RTW reduces with longer absence [6].

In Norway, employees typically receive full wage benefits for one-year during sick leave through the national insurance scheme administered by Norwegian Labour and Welfare Administration’s (NAV) [10]. If an employee does not resume work within one year, they may apply for a transitional benefit of ‘work assessment allowance’ (AAP), and typically become regarded as being ‘out of work’ [11]. In 2024, AAP was granted for up to three years for individuals undergoing health-related treatment, rehabilitation, or vocational actions [11]. Disability pension, a lifelong benefit, maybe Granted if NAV determines that the individual receiving AAP has at least 50% permanent incapacity for work. Individuals who have received disability pension tend to have undergone multiple failed healthcare and/or vocational interventions [12].

Among the working age population in Norway in 2023, 4.2% received AAP and 10.5% received disability pension [13, 14]. Approximately one-third were attributed to persistent pain [14]. Studies on RTW in pain populations generally focus on people on short and/or on people with specific diseases or disabilities within certain rehabilitation contexts [15]. Several studies have examined factors associated with prolonged sickness absence and disability due to musculoskeletal pain [16–19]. Perceived obstacles or facilitators of RTW have been studied from various perspectives, including employers [20, 21], workers [22–25], and significant others [21, 26]. While these studies provide useful insights, understanding experiences of individuals who are navigating the RTW process, particularly for those reliant on welfare benefits, may be instructive for the design of future interventions. Further, there is limited research in which both facilitators and obstacles have been considered together, especially RTW experiences of people on welfare benefits. Our aim was to explore obstacles and facilitators of RTW among individuals receiving welfare benefits (i.e., AAP and/or disability pension in a Norwegian context).

## Materials and methods

### Study setting and participants

Participants were recruited from a cohort randomised controlled study in Norway, in which RTW rates among unemployed people with persistent pain were being explored [27, 28]. Recruitment to the study was through social media and organisational newsletters Those eligible were aged between 18 and 64, unemployed for at least one month, had pain for more than three months, and wanted to work. Within the cohort study, participants consented to be approached for an interview study. Potential participants were purposively targeted by age, gender, pain duration, unemployment duration, and RTW status, based on six-month follow-up data from the cohort study [29]. The aim was to recruit approximately 15 participants for each group (RTW and no RTW), which was considered likely to be sufficient to approach ‘data saturation’ (i.e., the point beyond which relatively few novel experiences are being described) [30]. Author PA contacted potential participants by phone to inform and invite them to participate. To encourage recruitment, NOK 200 (approximately EUR 17) in universal gift cards was offered. Interested individuals were sent an invitation letter, participant information leaflet, and consent form. PA addressed any queries from participants before scheduling.

## Data collection

A semi-structured in-depth topic guide, developed with user representatives, was used to facilitate exploration of RTW obstacles and facilitators (Additional File 1). The topic guide and interviewing process were piloted on three individuals who were unfamiliar with the study, and the finalised topic guide encompassed themes related to pain, welfare systems, RTW attempts, and associated obstacles and/or facilitators. Interviews were conducted face-to-face at participant’s homes, or via video-link using Zoom (Zoom Video Communications, San Jose, California). In both instances, the ‘Nettskjema Diktafon-app’ (UiO, Oslo) was used for secure audio recording. Interviews were manually transcribed verbatim, and each transcript was assigned a unique participant identification number. Identifiable information was redacted. For face-to-face interviews, a consent form was provided and signed on-site, while a secure online consent process (Nettskjema; UiO, Oslo) was used for video-link interviews.

### Data analysis

Data were analysed using the Framework Method, a systematic approach to organise and analyse interview transcripts, supported by NVivo 12 (QSR International, Victoria, Australia) [31]. The method enables comparison and contrast of data by themes across multiple cases while retaining the connections with other aspects of individual accounts. The steps outlined by Ritchie and Lewis (2014) were followed [31]: familiarisation, identifying an initial framework, indexing, charting data into the framework matrix, and mapping and interpretation. Authors PA and PI independently coded a selection of transcripts, developing an initial framework. A third researcher contributed to discussions on the suitability of the initial framework and emerging themes, and possible meanings and interactions. PA and PI then agreed on the themes and used these to preliminary code. Indices were used for the remaining transcripts, revised if necessary, and the data were charted into a framework matrix [32]. The matrix provided an overview of each participant’s perceived obstacles and facilitators to help classify and organise data. Any challenges encountered during indexing and charting were documented and discussed, with changes being made to the framework as necessary. Further, the research team and user representatives engaged in discussions to interpret data and developed explanatory models to explain patterns between RTW status and experience of facilitators and obstacles within RTW journeys.

## Rigour

To provide a transparent audit trail from data collection through to interpretation, the interviewer kept journal records reflecting on their own thoughts and influences (reflexivity) for each interview [33]. Investigator triangulation was implemented during analysis, involving two researchers in the analytical processes to mitigate individual influences on data interpretation [34]. Additionally, presentations of the developing analysis and potential explanatory models were presented to peers and user representatives who assisted with interpretation and conceptualisation [35].

## Ethics

The study protocol was evaluated by the Southeast Regional Committee for Medical and Health Research Ethics as part of the cohort randomised approach (record no. 402918). A data protection impact assessment was conducted in collaboration with the Norwegian Centre for Research Data, and informed consent to participate was obtained from all participants in written form prior to enrolment.

## Results

Out of 38 people invited, 32 (84%) consented to participate. Three withdrew before interview, citing personal circumstances. Interviews were conducted between November and December 2023, and ranged in duration from 34 to 85 min with an average of 59 min. At point of interview, 12 participants had returned to work and 17 remained unemployed. Most participants were women (22 out of 29), and participants’ ages ranged from 27 to 63 years, averaging 41 (SD = 10). Participants lived across 13 out of 15 counties in Norway. Most had experienced pain for over 10 years and had been out of employment, on benefits, for at least two years (Table 1).

**Table 1:** Participant characteristics at baseline, with RTW status at time of interview

ID	Gender	Age group	Duration unemployed	Pain duration	Health scale^1^	Benefit status (FTE)^2, 3^	RTW status follow-up^2^
PQ04	F	30–39	> 2 years	> 10 years	71/100	N/A	RTW 100%
PQ07	F	50–59	1–2 years	3–10 years	35/100	AAP (50%)	RTW 50%
PQ08	F	60–64	> 2y years	> 10 years	41/100	DP (60–80%)	RTW 20–40%^**4**^
PQ12	F	40–49	> 2y years	> 10 years	70/100	DP (60–80%)	RTW 20–40%^**4**^
PQ14	F	40–49	1–3 months	> 10 years	16/100	DP (60–80%)	RTW 20–40%^**4**^
PQ15	F	30–39	> 2 years	3–10 years	10/100	DP (50%)	RTW 50%
PQ21	F	40–49	> 2 years	1–3 years	60/100	AAP (6%)	RTW 60%
PQ23	F	40–49	1–2 years	1–3 years	76/100	AAP (50%)	RTW 50%
PQ24	F	30–39	> 2 years	> 10 years	60/100	AAP (60%)	RTW 40%
PQ26	F	30–39	1–2 years	1–3 years	46/100	AAP (50%)	RTW 50%
PQ27	F	50–59	> 2 years	> 10 years	20/100	DP (60–80%)	RTW 20–40%^**4**^
PQ28	F	50–59	> 2 years	> 10 years	45/100	N/A	RTW 100%
PQ01	M	30–39	> 2 years	3–10 years	60/100	DP (100%)	No RTW
PQ02	F	50–59	> 2 years	> 10 years	38/100	DP (100%)	No RTW
PQ03	F	50–59	> 2 years	> 10 years	70/100	DP (100%)	No RTW
PQ05	M	30–39	> 2 years	3–10 years	20/100	DP (100%)	No RTW
PQ06	M	40–49	> 2 years	> 10 years	30/100	DP (100%)	No RTW
PQ09	M	20–29	> 2 years	3–10 years	30/100	DP (100%)	No RTW
PQ10	M	40–49	> 2 years	> 10 years	38/100	DP (100%)	No RTW^**5**^
PQ11	F	30–39	6–12 months	1–3 years	20/100	AAP (100%)	No RTW
PQ13	M	60–64	> 2 years	> 10 years	31/100	DP (100%)	No RTW^**5**^
PQ16	F	50–59	> 2 years	1–3 years	80/100	AAP (100%	No RTW
PQ17	F	50–59	1–2 years	3–10 years	78/100	AAP/DP (50/50%)	No RTW
PQ18	F	30–39	> 2 years	1–3 years	40/100	AAP (100%)	No RTW
PQ19	F	40–49	> 2 years	3–10 years	20/100	DP (100%)	No RTW
PQ20	F	40–49	> 2 years	> 10 years	40/100	DP (100%)	No RTW
PQ22	M	50–59	> 2 years	3–10 years	70/100	DP (100%)	No RTW
PQ25	F	30–39	> 2 years	> 10 years	35/100	DP (100%)	No RTW
PQ29	F	50–59	1–2 years	1–3 years	30/100	AAP (100%)	No RTW

^1^ EQ visual analogue scale (EQ VAS, 0 to 100) from the EQ-5D-3 L instrument: 0 = worst imaginable health state, 100 = best imaginable health state (Hansen et al., 2020)

^2^ Based on six months follow-up data

^**3**^ AAP = Work Assessment Allowance, DP = Disability Pension

^**4**^ Varying percentage based on on-call duties

^**5**^ Stated to sometimes work up to tax-free income limit (< 10% FTE)

Transcripts were carefully reviewed by PA and PI, before six transcripts were independently coded. After familiarisation, data interpretation led to the consensus to inductively code transcripts, regardless of participants’ RTW status, into: (1) obstacles to RTW, and (2) facilitators to RTW. During analysis, the same themes emerged under ‘obstacles’ and ‘facilitators’. These related to: 1) Norwegian Labour and Welfare Administration, 2) healthcare, (3) psychological, (4) pain, (5) perceptions of work, apart from ‘insurance’ (obstacle), and ‘luck’ and ‘disability pension’ (facilitators). A total of 24 sub-themes were coded under obstacles and 18 under facilitators (Additional File 2). Generally, participants emphasised obstacles related to NAV and the public healthcare system, while facilitators were often described as fortunate encounters or events within the systems. Quotes typifying what has been described in themes have been translated into English by the researchers. Original Norwegian quotes and supplementary quotes for each subtheme are found in Additional File 3.

### Perceived Obstacles and facilitators to return to work: explanatory model

**Fig. 1:**
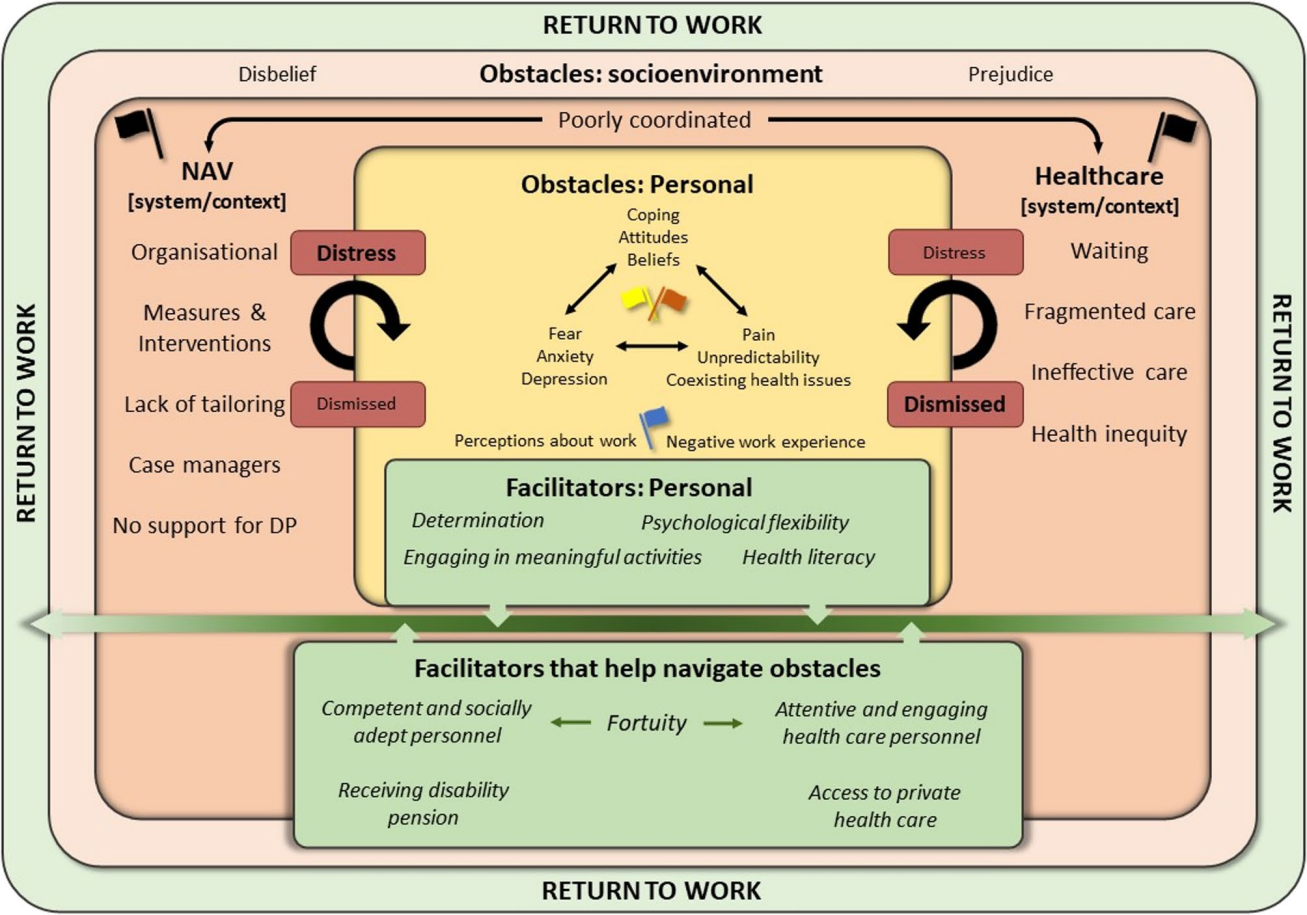
Explanatory model of obstacles and facilitators of return to work. Participants (personal) describes being embedded within NAV and healthcare (systems/context), with facilitators primarily helping to navigate obstacles towards return to work.

Participants described that obstacles and facilitators were present in NAV and healthcare systems, alongside overarching socioenvironmental prejudice and disbelief (Fig. 1). The perceptions encompassed stigma related to an affiliation with NAV, unclear persistent pain symptoms, and coexisting health issues (Q1.54, Additional File 3). Descriptions related not only to their close social environment - including friends, family, and pervious colleagues – but also to the NAV and healthcare systems (Q1.23). The reported obstacles can be understood through the Psychosocial Flags Framework, which categorises psychosocial obstacles into yellow, blue, and black flags [36]. Yellow concern the person’s psychosocial factors, blue concern workplace perceptions, black involve contextual systems and policies, and orange flags indicate psychiatric symptoms such as depression and anxiety [37].

Overall, participants described a NAV system that they judged not organised to assist people with persistent pain returning to work (Q1.16). They asserted that NAV created obstacles such as lack of tailored support, emphasis on ‘testing’, and distress associated with documentation, lack of belief, financial worries, and fear of consequences (Q1.22). Participants indicated that they thought the NAV structure and organisation did not sufficiently consider individual needs and health issues (Q1.18). Participants experienced long waiting times within the healthcare system (Q1.83), and GPs with limited intervention options (Q1.95). In general, participants thought that persistent pain was inadequately managed and often dismissed (Q1.96). Participants emphasised perception of fragmentation within the healthcare system, which focused on single health issues, despite persistent pain often being accompanied by mental health comorbidities (Q1.87). Participants believed there was insufficient collaboration between NAV and the healthcare system (Q1.7), perpetuating cyclical actions within both systems; such as repeated work-ability assessments (Q1.19), or continuous healthcare referrals initiated by GPs (Q1.97). This resulted in a pervasive sense of disregarded concerns and distress (Q1.98).

Nearly all participants reported experiencing pressure, stress, and distress associated with NAV involvement, as the focus remained solely on RTW, ‘work testing’ and documentation, rather than on their health concerns and overall well-being (Q1.21), which significantly influenced individuals’ psychological well-being, pain experiences, coping mechanisms, and belief systems (Q1.41). In general, those who successfully returned to work appeared to report fewer issues related to pain and coexisting health issues, and talked more about determination, health literacy, empowerment, and psychological flexibility (Q2.34). A divergence in the RTW narratives is related to encounters with individuals within NAV or the healthcare system, who were seen as attentive, engaging, and socially adept, in addition to possessing perceived competence and a willingness to ‘bend the rules’ (Q2.68). These fortunate encounters, combined with personal facilitators, helped some participants navigate systemic and contextual obstacles to work. Furthermore, within the NAV framework, receiving a disability pension was described as a relief, as it diminishes obstacles associated with NAV (Q2.73). Having the financial means to access private healthcare also mitigated some of the obstacles related to healthcare, particularly waiting lists and health inequities (Q2.64).

### Perceived obstacles and facilitators to return to work: description of themes and example quotes

#### Norwegian labour and welfare system (NAV) - perceived Obstacles and facilitators

Participants reported experiencing a range of obstacles within the NAV system that affected their perception of receiving adequate support for their health issues and RTW efforts including complex and rigid rules, difficulties interpreting documents and decisions, frequent changes of case managers, and long waiting times.

*“Because I felt like every time I got a NAV case manager*,* I had to go through the whole story again. They could come to a meeting without having looked at your file. So you had to start all over again*,* every time. And every time they had a new explanation about what we should do. Because then they come up with new ideas every time.”* - PQ02.

A lack of information about opportunities, rights, and application deadlines was reported as typical and participants reported experiencing challenges related to what they perceived as case managers’ lack of competence, understanding, empathy, and care, and that the case managers viewed them as ‘numbers in a system’ rather than individuals.

*“Here it’s all about figures and money and numbers. I don’t know*,* it feels like that. You’re just a number in line. You’re not a person who’s sitting there with health problems initially.”* **- **PQ03.

*“Because this NAV case manager*,* even when you’re lying there vomiting from pain*,* still wants to force you into testing and clarification. When*,* in the first place*,* you might deserve to be allowed to have a little peace at first.”* - PQ27.

Participants reported thinking that NAV measures were not adequately tailored to address their pain issues, and that they perceived a lack of understanding regarding the relationship between physical and mental health. Participants often shared experiences of stress, fear and frustration, anger, and degrading encounters in their interactions with NAV.

*“They group together people who struggle to get a job*,* because they don’t know how to get a job*,* and those who are sick. And then they put everyone through the same process. I don’t need to know how to write a resume or a cover letter*,* or*,* yeah… It was not matched with my needs… to get some guidance. But that type of help… there was no competence or access to it.”* - PQ07

*“It’s fear. I could be dreading a NAV meeting for a whole week in advance. It was absolutely terrible. I would go with heart palpitations and feeling nauseous. It’s absolutely horrible. Migraine attacks and everything. Because your whole body tenses up. When you are constantly being threatened that they will take away your benefits*,* what do you do then? You are completely dependent on them. I found it incredibly tough”.–* PQ02.

Participants receiving disability pension reported experience a lack of support from NAV; perceiving that they were no longer a ‘part of the team’.

*“You could have thought that NAV could support you…or support me in getting back to work. But they say*,* no*,* you are completely disabled and cleared*,* and then that’s true.”* **– **PQ14.

“*When you become a disability pensioner*,* that’s the end of it. The only thing I have now is my regular doctor. And all he can do for me is prescribe painkillers.”* **– **PQ19.

Some reported positive experiences. A precise diagnosis, such as rheumatoid arthritis, simplified interactions, compared to non-specific pain issues. Participants in rural districts mentioned stability from dealing with fewer case managers. Some mentioned being allocated a supportive and competent case manager; one able to adapt to their needs, explain processes, use shared decision-making, and encourage trial and error. However, they thought this a matter of ‘luck’.

*“Then she said*,* “Now you will get one year*,* where you can get what you need.” And I was startled. “If you want additional education*,* further training*,* whatever. So that you can return to work”. When I left her office*,* I cried. And I told her*,* “If you see someone jumping and dancing down the street*,* that’s going to be me.” Because I got what I needed*,* and I was back at work a year later*.” - PQ08.

Participants valued case managers and managers in external work placements who displayed flexibility, understanding, engagement and the ability to ‘find loopholes’ within regulations or reporting requirements. Re-education, skill enhancement, gradual starts in work placements, and wage subsidies was experienced as facilitators.

*“If I hadn’t received the education I did*,* I might not have had that opportunity. I might have had to work less*,* perhaps being partially on sick leave or something similar. So*,* I am very grateful that NAV helped me get on the path of retraining.”* - PQ04.

*“I got the feeling that she genuinely wanted to help me and shielded me from things that would harm rather than help me. So*,* it was very… and I was completely turned around because I went in with such negative experiences that had pretty much shaped me. So*,* I was very pleasantly surprised*,* and she has been the one who has helped me move forward. And it is because of her that I am where I am today.”* - PQ07.

#### Healthcare system - perceived Obstacles and facilitators

Most participants described frustration around long waiting times for examinations, diagnosis, or treatment. Healthcare services were described as fragmented, with multiple referrals to specialist healthcare services, each accompanied by long waiting times.

*“What breaks most people is the waiting time… that’s what it is.”* – PQ16.

*“Firstly*,* it’s the healthcare system. It just takes such a long time before you get… it took a good six months before I saw a specialist.”* – PQ17.

Participants discussed feelings of being rejected by specialist services, particularly regarding mental health problems. Some also believed that surgery had worsened their pain symptoms, or that surgery was attempted as an inappropriate last resort. Others had tried multiple different ’out-of-pocket’ treatments, out of desperation, or due to long waiting lists in the public healthcare service.

*“Even when I’ve gone to the emergency room and cried*,* they’ve referred me to an urgent appointment. And at the urgent appointment*,* I’ve been clearly told that they can’t help me because I don’t have psychosis. So*,* in a way*,* I have to be extremely down to get help from the DPS* [District Psychiatric Center] *or other institutions offering mental health support.”* – PQ11.

*“I have tried different therapists. I have tried different therapies. I go for a while*,* three*,* four*,* five months. No improvement. In most of the therapies I started*,* the focus was only on the pain. I give up.”* – PQ19.

Some were prescribed opioids, which they thought was due to their GP having a lack of treatment options. In some cases, participants thought this led to unwanted side effects and may have led to dependency.

*“But I felt like they threw a lot of pills at me. That was unfortunate. Especially since I was so young. I was only about 20. I got fifty morphine tablets a week. It was quite a lot. It was hard to quit.”* – PQ04.

Participants thought that, in general, healthcare providers lacked knowledge about persistent pain and did not appreciate how it related to physical and mental health. Some suspected that they had been misdiagnosed after medical examinations. Many stated that they had experienced prejudices and felt dismissed or not taken seriously, especially when medical examinations came back ‘negative.’ They thought that their symptoms were being trivialised, with some finding provided explanations to be condescending, and designed to give the impression that their condition was ‘all in their head’.

*“He thought that my problems were psychological*,* and I was like*,* no*,* but you become very mentally worn out from being in pain all the time*,* being tired all the time*,* and not having money.”* – PQ25.

*“The worst are those who work in the healthcare system. I’ve heard doctors say*,* “No*,* but you see*,* here we deal with life and death.” Or… “Well*,* well*,* we have to learn to live with the little things.” Comments like that*,* but it’s mostly the healthcare system. Because then everything accumulated—the disbelief*,* no hope*,* nothing that could be done*,* and it was all about how you handle it. Now you just have to smile and be happy*,* and it’ll be fine. And that wasn’t a solution.”* – PQ07.

Some participants described thinking their GP is overworked and believed that the referral to other services was a way to ‘transfer the problem to someone else’, leading to cyclical referrals. A lack of cooperation between NAV and GPs was perceived, along with a sense that NAV did not trust GPs’ advice. This lack of cooperation increased the frustrations and challenges for participants.

*“The general practitioner*,* you know*,* they’re just like I said about NAV*,* they’re overwhelmed with work*,* right*,* so everything is in and out*,* in and out*,* so it kind of falls on you then.”* – PQ05.

*“When it comes to diffuse issues*,* you’re kind of cast aside in the healthcare system. Because not all pain can be explained.”* – PQ27.

Some participants accessed healthcare that they considered was aligned with needs, contributing positively to their overall well-being. This included services like counselling, group sessions, cognitive therapy, and various complementary treatments such as chiropractic, osteopathy, and physical therapy.

*“And then I was referred to the pain clinic in* [anon]. *It was only then that I truly got proper help. That was really when I felt like*,* okay*,* now I am getting proper help.”* – PQ14.

*“I attended a rehabilitation programme at* [anon] *two years ago*,* and it was a very positive experience because everyone was in the same… How should I put it? Everyone was in the same boat. Everyone had their own burdens. We didn’t talk about problems and illnesses and such. There was a reason we were there*,* whether we saw it or not. It was a very positive experience to be among like-minded individuals*,* even though we were all different.”* – PQ21.

Others mentioned that insurance or personal funds allowed them to access healthcare in private settings, reduced waiting times for examinations, and treatments. For a few, successful surgeries were described as relieving the worst pain symptoms.

*“What has helped me is that my employers had insurance for me. That way*,* I can get an appointment with a neurologist or rheumatologist within three days. Even when I needed surgery*,* I was told that they would schedule the operation*,* and I could get an appointment in a week.”* – PQ15.

Some discussed valuable support they received from engaged and dedicated healthcare professionals. Some GPs were highlighted as invaluable due to their competence in dealing with persistent pain issues, as well as their ability to listen and motivate.

*“I have a very good doctor who is very understanding and very good when it comes to pain. He has competence regarding pain. So*,* he and I have agreed that the most important thing is that I have a quality of life. To stabilise the pain so that I have a quality of life*,* that I am able to work a little*,* go out for walks. Basically*,* to be able to feel good.”* – PQ12.

#### Psychologically related - perceived Obstacles and facilitators

Participants discussed several obstacles related to their mental and emotional health, including poor pain coping strategies and negative attitudes and beliefs. Many reported experiencing depression, anxiety, and fear directly resulting from their interactions with NAV processes.

***“****I have been very down at times*,* so… so far down that I tried to end it all. I’ve been down there many times. And that’s because the pain has become so intense*,* and I’ve faced so much adversity both with NAV and the healthcare system”* – PQ14.

***“****I think it’s very easy*,* when you get sick yourself*,* to become scared. Pain makes you scared. And then it’s very easy to retreat into that bubble and feel sorry for yourself.”* – PQ27.

Other obstacles included challenges of chasing diagnoses, receiving unclear diagnoses, coexisting ailments, severe pain, and financial concerns. These led to negative and pessimistic thoughts about themselves, and their quality of life and future. Unfavourable self-management practices were also reported, such as the overuse of pain medication and overexertion, and participants drew their own further inferences from these factors.

***“****But what happens is that I push myself too far because I find it so enjoyable. And then I start to feel pain*,* but I just ignore it*,* and eventually*,* I can hardly stand on my feet.”* – PQ08.

*“So*,* it’s probably quite a lot of medication then. Yes*,* it is. I originally thought that the more I took*,* the less inflammation there would be in my body and the better I would feel. But it turns out that I’ve likely been taking way too much for a long time. And that has affected my mind and everything else.”* – PQ06.

Several expressed feelings of frustration, anger, and hopelessness over not being believed by other people, healthcare professionals, and NAV. This lack of understanding appeared to lead to emotional distress. Some also noted a lack of support from friends and family, which exacerbated their sense of isolation.

***“****And then there’s this thing with society… like with taking walks. You can manage to go for a walk*,* but you can’t manage to go to work. You can travel on vacation*,* but you can’t manage to travel to work. You feel like you have to constantly defend yourself just to live.”* – PQ21.

***“****But there are many*,* doctors who have told me*,* both at the hospital and other doctors have said to me*,* that the pain is psychological.”* – PQ15.

Psychologically oriented facilitators primarily involved maintaining a positive and forward-looking attitude. This included setting personal goals, developing self-awareness, understanding their own limitations, and engaging in self-management.

*“I think it must be that I am generally quite positive. Instead of seeing problems*,* I mostly see solutions. It doesn’t occur to me to give up in a way*,* because things always work out.”* – PQ24.

*“I think that when you’ve been at home for a while*,* and you don’t have any money*,* you don’t have the energy*,* you don’t have the desire*,* you’re just in pain and exhausted and losing hope*,* taking steps towards a goal is so important.”* – PQ04.

Several described a shift in mindset, which involved accepting their changed life situation, leading to a more adaptive and proactive approach, with some highlighting their willingness to explore new opportunities, such as pursuing re-education or training for a different career. They also aligned their original ‘job-identity’ to their current limitations and were open to try out activities and strategies recommended by healthcare professionals or NAV case managers.

*“So I used to be a career woman. It’s quite a big step from doing that*,* to working in a [anon]*,* but that’s where I need to be now.”* – PQ07.

*“…so I go and exercise*,* no matter how bad I feel… it’s almost like I always go and exercise. And when I say exercise*,* it’s not like how I used to say it before. It’s just about getting out there; it’s for the sake of my mental health.”* – PQ05.

Knowledge about health – how the body and mind work when experiencing pain – was expressed as a facilitator for forming questions, seeking opportunities, and finding solutions. Additionally, social support from key people and a strong willingness to achieve targeted goals were reported as important. Engaging in voluntary work was reported as providing significant meaning and increased quality of life.

*“I think it’s a bit about not being so scared anymore. “If it feels like this*,* it might hurt. Is this a good idea? Am I doing this correctly now? Will it get worse? Is this smart? I can handle this. My body is strong*,* it can handle this. Everything is good*,* everything is fine. If it gets worse*,* I’ll take it easier next time. Relax a bit more. There’s nothing structurally wrong with my body.” And then I remind myself of that. That I am healthy*,* I am strong*,* I am safe*,* and it’s going to be okay. That has probably had some impact on feeling stronger and approaching exercises in a slightly different way.”* – PQ23.

*“Like volunteer work… Trying to be a bit engaged and thinking that I have to somehow live with my limitations. But I believe that shouldn’t stop me from doing everything. So*,* I think it’s partly that I don’t want to just stay at home.”* – PQ12.

#### Pain related - perceived Obstacles and facilitators

Several participants reported co-existing symptoms or health issues, both physical and psychological. These symptoms and coexisting health issues were described as unpredictable and creating significant obstacles to participating in working life.

*“And eventually the fatigue*,* because it was really the fatigue that became the biggest problem over time. I think the pain… in the worst case*,* I can live with it. But being fatigued*,* that’s*,* that’s torture. I think it’s worse than being in pain.”* – PQ25.

These health issues were experienced as affecting functioning and capacity, creating uncertainty about how to distribute their energy - e.g., whether to focus on work or home life. Participants also talked about challenges posed by poor sleep.

*“When I*,* for example*,* explain that I can spend an entire day to a whole week getting housework done*,* or when I talk about all the things I can’t manage… or that I can go to work*,* but when I come home*,* I am completely exhausted for the rest of the day.”* – PQ18.

*“Because I struggle a lot with sleep. Because I don’t… I’m in so much pain. That you… You can’t fall asleep*,* and then you wake up again because it hurts. And then you are completely foggy in the morning.”* – PQ12.

A few mentioned that their pain or coexisting health issues had slightly decreased or become more stable over time, either through self-management or by receiving beneficial treatments and interventions.

*“And since I still have pain*,* there is some form of nerve pain related to it. Because my pain levels have become much more stable after I had surgery.”* – PQ07.

#### Work related - perceived Obstacles and facilitators

Some participants considered that the labour market is not set up to accommodate people with health issues, particularly in terms of adjustments and flexibility. These participants recounted previous negative experiences during sick leave or work-placement, including lack of willingness to offer adjustments, pressure from managers or colleagues to be present and effective, and instances of social exclusion.

*“Because I heard that the others have to work more to make up for you. Because you’re not here. And that’s not nice to hear.”* – PQ02.

Some had positive experiences from work placements or current employment (i.e., within those who had returned to work), such as workplaces in which a willingness to be flexible and adaptive was demonstrated and people had a genuine interest in supporting them.

*“So*,* both the work tasks and the work environment… meaning that I had a centred roller mouse and a proper chair and a sit-stand desk*,* and all this*,* right*,* that things were adapted*,* and you had the opportunity to take a break and go for a walk*,* or sit down on another chair*,* or something like that. When it was accommodated*,* it worked well.” –* PQ21.

#### Other perceived obstacles

Less discussed obstacles were insurance issues and a lack of financial incentives to work. Some of those receiving disability pension perceived few incentives to RTW, as benefits are reduced if the income exceeds a set limit, which was perceived to be very low. Some noted that with private disability insurance, the support/payments disappear as soon as the first salary is received. This led to concerns, and a reluctance to take the risk of returning to work given the unpredictability of their health.

*“From the first earned krone* [NOK], *in my case*,* I lose my insurance money*,* which is* [NOK] *5000 kroner a month*,* plus the child supplement for my disability*,* which is* [NOK] *4700 kroner. So that means I lose* [NOK] *10*,*000 kroner. If I start working again*,* no matter the percentage*,* I lose it. And what I mean by that is*,* of course*,* I want to work*,* but that one day could cost me so much.” –* PQ05.

Insurance conflicts were related to compensations awarded for permanent physical or psychological impairments that participants believed were the result of a work-related injury or illness. Conflicts with Norwegian Patient Injury Compensation (NPE) were also discussed; specifically, participants noted that in order to receive any compensation from NPE, participants had to prove that something went wrong during diagnosis or treatment.

*“I can say that I have had my battles with the insurance company*,* because it has been an insurance matter.” –* PQ14.

#### Other perceived facilitators

Participants expressed that being fortunate played a significant role in meeting the ‘right type of person’ within the healthcare system, NAV system, or through NAV-contracted work-placements. They felt lucky to have had a GP or case manager who provided valuable support or a previous employer that provided health/disability insurance.

*“Then I got to try a new work-placement through* [anon]. *And the woman at* [anon], *she was absolutely fantastic. It was… It’s perhaps… I mean*,* without her*,* I wouldn’t be where I am now” –* PQ15.

*“Well*,* it’s NAV that pays for these… But I was lucky this time to… met someone… and sometimes you are lucky… She was someone who managed to… that she maybe had enough clout to dare to… not just put me through the NAV grinder*,* because they need to check boxes and write reports to NAV.” –* PQ07.

Being granted disability benefits was perceived as positive by most participants. Such participants spoke about a sense of freedom and re-gaining control over their lives: the reduction in financial worries allowed them to focus on recovery and well-being.

*“But when she said that you can go back to work again*,* it was in the old days that when you became disabled*,* you were done. But when I finally sort of understood that it would calm me down so much. Then everything with the reporting cards and all that would become easier because you don’t have to deal with that. And that was absolutely true.” –* PQ03.

These participants emphasised that disability benefits provided them with more time and energy to assist family and friends, contributing at home or in the community. They experienced peace of mind, allowing them to consider the future and the possibility of returning to work, and a feeling that ‘the struggle is over.’ Additionally, they were free from the burden of reporting and maintaining a dialogue with NAV, as well as from unhelpful but mandated measures and interventions.

*“So he was very much about the fact that you gain a bit of surplus which you are in control of. You don’t have NAV over you. You don’t have anyone who is going to test you out. So that’s something that can help. I’ve tried to take that to heart*,* that now it’s basically just me. And now I control myself. And in a way*,* what I want*,* nobody is coming to monitor me. I am not on any measures. So I think that helps.” –* PQ12.

*“And perhaps get myself some jobs eventually*,* and build that up. One advantage of disability benefits is that I don’t have to have contact with NAV.” –* PQ08.

#### The complexity of return to work: a game of snakes and ladders

**Fig. 2:**
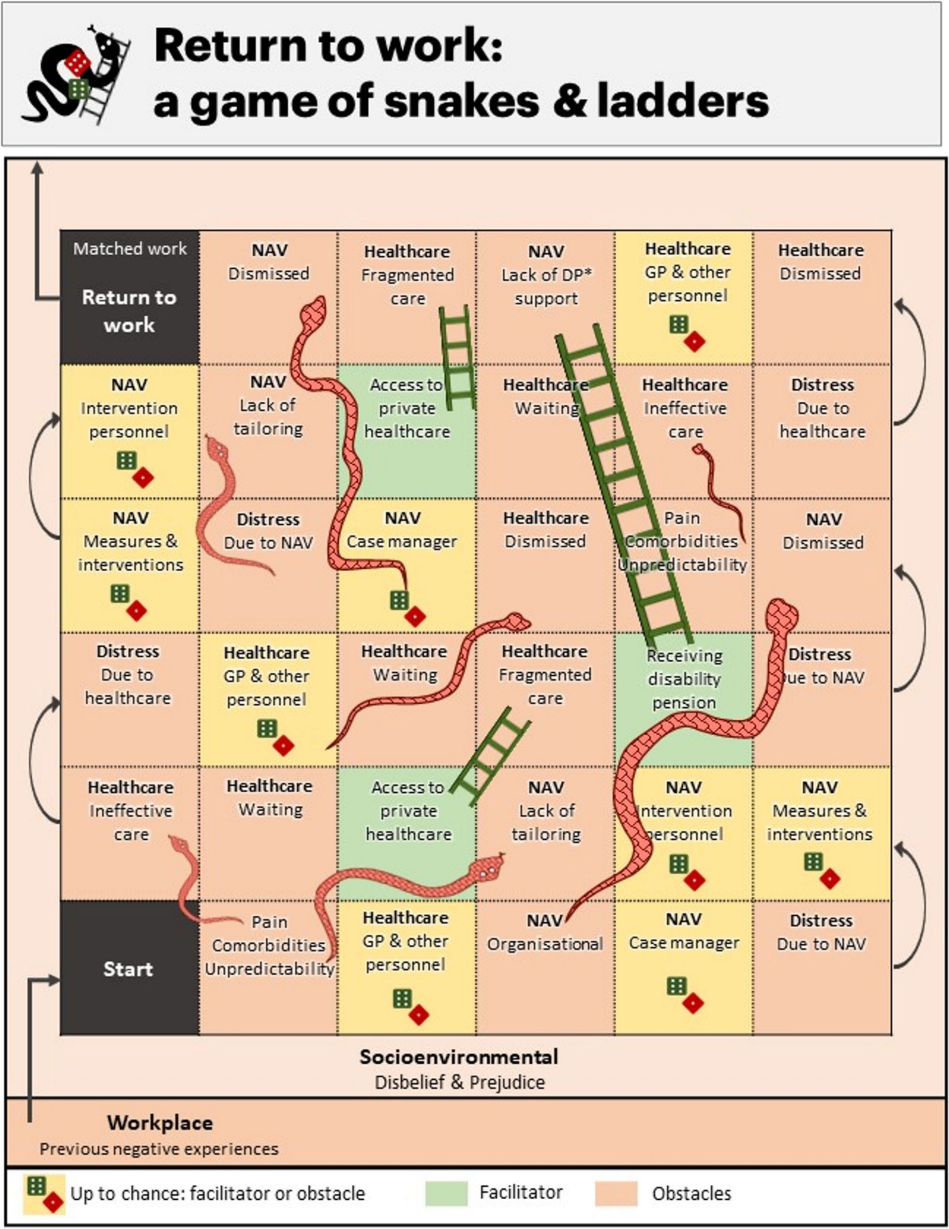
Return to work while struggling with persistent pain: contextualising the journey in a game of snakes and ladders.

Developed in collaboration with user representatives, exploring the relationships in participants’ narratives of their RTW journey might be allegorically likened to playing a game of ‘Snakes and Ladders’ (Fig. 2).

Here, the dice represent chance encounters with a helpful facilitator or discouraging obstacle. For some, snakes came early and frequently; long waits for healthcare services, repeated work-ability assessments from NAV, and dismissive attitudes towards their persistent pain and coexisting health issues. Each snake encountered took them back, inducing stress, pressure, and a sense of being disregarded. However, for those who managed to climb ladders, the journey had been punctuated by moments of luck; fortuitous interactions with compassionate, competent professionals who provided the bit of extra support needed. Ladders helped bridge the systemic and contextual gaps, moving them closer to the goal of RTW. Social support, and personal qualities like determination, health literacy, psychological flexibility, and sometimes the financial means to access private healthcare, acted as internal ladders, enabling self-efficacy and facilitating a climb past multiple snakes. In contrast to the distressing steps back, achieving a disability pension gave people a sense of reaching a safe patch, where the constraints of NAV were lifted, and financial stability allowed them to rest and regain some control over their life’s direction. In this game of chance, ‘winning’ is marked by RTW, while obtaining a disability pension provided stability and relief as ‘pausing’ the game. Some were being caught in a perpetual loop of setbacks, assessments, and feeling ignored or dismissed by a fragmented system. The fortunate dice rolls amidst a challenging landscape of obstacles, depicts a thin line between successful outcomes and distressful journeys in a complex game of navigating life’s challenges while managing persistent pain.

## Discussion

Participants described their perception of a welfare system that is inadequately organised to help them to RTW. They considered NAV to be a major obstacle between them and both RTW and better health; especially in respect of perceptions of a lack of tailored support, and an emphasis on work ability testing and documentation, which led to fear and distress. The healthcare system was perceived as fragmented, with long waiting times, limited intervention options, and a tendency to dismiss persistent pain and coexisting mental health issues. Insufficient collaboration between NAV and the healthcare system was described as perpetuating a cycle of repeated assessments and referrals, compounding distress. Despite perceptions of such systemic and contextual obstacles, some managed to RTW. Descriptions of fortunate encounters with attentive and competent individuals within NAV or the healthcare system, along with positive psychological traits, helped some to navigate obstacles. Receiving a disability pension was thought to provide relief from major NAV-related obstacles, allowing individuals to shift their focus from mandatory, yet seemingly unhelpful activities, towards recovery.

That participants espoused views that the current welfare system not only fails to address the needs of individuals with persistent pain but also acts as an obstacle, complicating recovery and RTW, raises several issues. One issue was the lack of tailored support with an emphasis on work ability testing and documentation. Implementing more personalised support mechanism within NAV can reduce the distress and create a more supportive environment that encourages RTW. This would require the time and resources of case managers in addition to training within the Psychosocial flags’ framework, and such provision, of more empathetic and individualised support, may improve RTW processes and outcomes. Another issue is the sense of insufficient collaboration between NAV and the healthcare services. It may be axiomatic that better integration between and within these services would facilitate pathways and may help to reduce distress and improve user perception of management and effectiveness. Receiving disability pension was described in terms of providing ‘relief’ from NAV-related obstacles; especially related to distress concerning repeated work ability testing, documentation, and mandatory activities.

Currently, most individuals reaching the end of their one-year sick-leave transition to AAP which is a ‘catch all’ benefit being described by our participants as lacking tailored support. Given the descriptions of system and contextual obstacles, consideration might be given as to whether it might be more effective to replace AAP with an alternative benefit and intervention pathway tailored for those with persistent pain. For example, one might consider a trial (in collaboration with NAV) of an intervention that contains the following features: (1) tailored support considering both physical and mental health aspects, (2) shared decision making, (3) a lower burden of documentation, (4) a predictable benefit payment, (5) integrated work and health services, (6) empathic case management from trained personnel, and (7) individualised work-placements focused on ability rather than testing disability. The conjecture being that such a pathway may allow individuals the time to focus their attention and capacity on recovery, while receiving person-centred and integrated health and work care. This, in turn, improves quality of life and RTW potential. At the time of writing, there is some evidence that supported employment interventions may help outside mental health conditions [38]. A similar trial, without items 3 and 4, is currently ongoing [27].

The reported system and contextual obstacles to RTW are likely to require a coordinated effort across individual, organisational and legislation levels [39, 40]. Further research is required to determine how best to overcome obstacles within the welfare system at different levels. The perceptions of people with persistent pain reported in this study may be useful to policy makers, or could be used to inform an independent multidisciplinary taskforce with public and patient involvement to suggest an outline of a new benefit and pathway for people unemployed due to persistent pain. The feasibility of an individualised pathway with a lower burden of documentation, and trained case managers acting as supportive collaborators and coordinators of ‘work and health’ for people with persistent pain (*vide supra*) could be tested in a NAV setting. Further research with NAV might explore new ways of supporting those receiving disability benefits back to work. For example, exploring whether supporting part-time or flexible arrangements, at least initially, may facilitate RTW.

A Norwegian study in which disability pensioners were interviewed, described similar experiences to those which are reported in our study in terms of inadequate support, inflexibility and insensitivity to individual needs by social security and work offices [5]. This lack of individualisation is further highlighted from the NAV perspective, were case managers find it challenging to follow up individuals sick-listed with non-specific musculoskeletal disorders due to the lack of a visible or specific diagnosis [41]. Similarly, other studies have highlighted perceptions of a bureaucratic and complex nature of benefit systems [25, 42].

Perceptions of inadequate interactions with an impersonal healthcare system, characterised by a lack of recognition and struggling to be believed and taken seriously, have also been described elsewhere [43–45]. Specifically, Gjesdal et al., describes two contrasting themes as “Feeling acknowledged as a person in the healthcare services” and “Feeling neglected as a person in the healthcare services” [43]. These echo our findings that some had fortunate encounters within the healthcare system. Furthermore, Klanghed et al., illustrate positive and negative encounters with healthcare and social insurance professionals as decisive for the RTW process [46]. Unsupportive encounters, for example, where participants report feeling disbelieved and accused of exaggerating or imagining their illness, are consistent with findings from previous studies [22, 45, 47]. This contrasts with individuals’ need to be understood and their expectations that health professionals will comprehend their pain and life circumstances [48].

Other healthcare obstacles expressed, such as long waiting times for appointments, or feelings of being caught in a cycle of repeated consultations, has also been described [22, 25]. There are notable similarities between the descriptions of a ‘changed mindset’ and ‘psychological flexibility’ among those who returned to work in our study, and the descriptions of successful RTW outcomes in other studies [47, 49]. These studies suggest people began to perceive pain as ‘the new normal’ and held ‘positive expectations concerning work attempts’. This is further supported by the description from Edén et al., who described disability pensioners sucessfully returning to work as ‘Go-getters’ with strong devotion to work, positive expectations and determination [49].

### Strengths and limitations

Both individuals who had returned to work and those who had not were included, allowing for comparisons between these groups. Interviewed participants were from 13 out of 15 counties in Norway, capturing a range of experiences and perspectives. The potential impact of the researchers’ roles during interviewing, data analysis and interpretation of data is acknowledged. It is possible that our backgrounds (see author biography) may have influenced the research processes; however, it was our intention to conduct a rigorous and reflexive analysis, balancing self-awareness with a primary focus on the interview data. The rigour of our research was enhanced through triangulation, wherein two researchers collaboratively conducted the data analysis and selection of themes for presentation, with additional insights provided by an independent third researcher.

Participants in our study were predominantly female (76%), which aligns with the gender distribution commonly observed in research on the unemployed and sick-listed pain population in Norway [50, 51]. Notably, five out of the six individuals who declined to be interviewed were male, although no explicit reasons were provided for their refusal. While the proportion of females with persistent pain in Norway is estimated to be around 58%, the overrepresentation in our sample (which was drawn from a cohort randomised study) remains unclear [52–54]. The gender imbalance may have implications for the transferability of our findings: specific challenges and experiences of males are underrepresented, and our results reflect more the female experience of the RTW processes.

Participants were purposively sampled from a cohort study that, while not its aim, primarily recruited people through social media platforms (e.g., within a ‘AAP’ Facebook group). The views of people actively engaging on social media platforms may differ from those who are not active in such Facebook groups, often being more politically active [55]. Our study focuses on subjective experiences and interpretations of personal and social circumstances; potential inaccuracies in recollection of prior experiences may exist, as participants had received benefits for between one and ten years.

## Conclusion

Returning to work with persistent pain is complex process. Participants described several common obstacles, regardless of whether they have returned to work or not, which appear to stem from the systemic and contextual factors within the public welfare system and NAV in particular. Reported obstacles include a lack of tailored support for persistent pain and distress associated with system interactions. Facilitators described by those who returned to work typically involved encountering a supportive case manager or healthcare provider and experiencing a shift in outlook towards acceptance and determination to progress. A dedicated, integrated, empathetic and person-centred pathway within the welfare system may better support individuals with persistent pain.

## Supplementary Information

Supplementary Material 1.

Supplementary Material 2.

Supplementary Material 3.

## Data Availability

The datasets used and/or analysed during the current study are available from the corresponding author on reasonable request.

## Abbreviations

AAP: Work Assessment Allowance
GP: General Practitioner
NAV: Norwegian Labour and Welfare Administration
RTW: Return-to-work
