# Supplementary figures and images for: Obstacles and facilitators of return to work among people with persistent pain who receive benefit payments: an in-depth interview study
Source: BMC Public Health. 2025 Oct 21;25:3532. doi: 10.1186/s12889-025-24747-0 (PMC12539112; doi:10.1186/s12889-025-24747-0)

## Supplementary file 2. Themes and subthemes

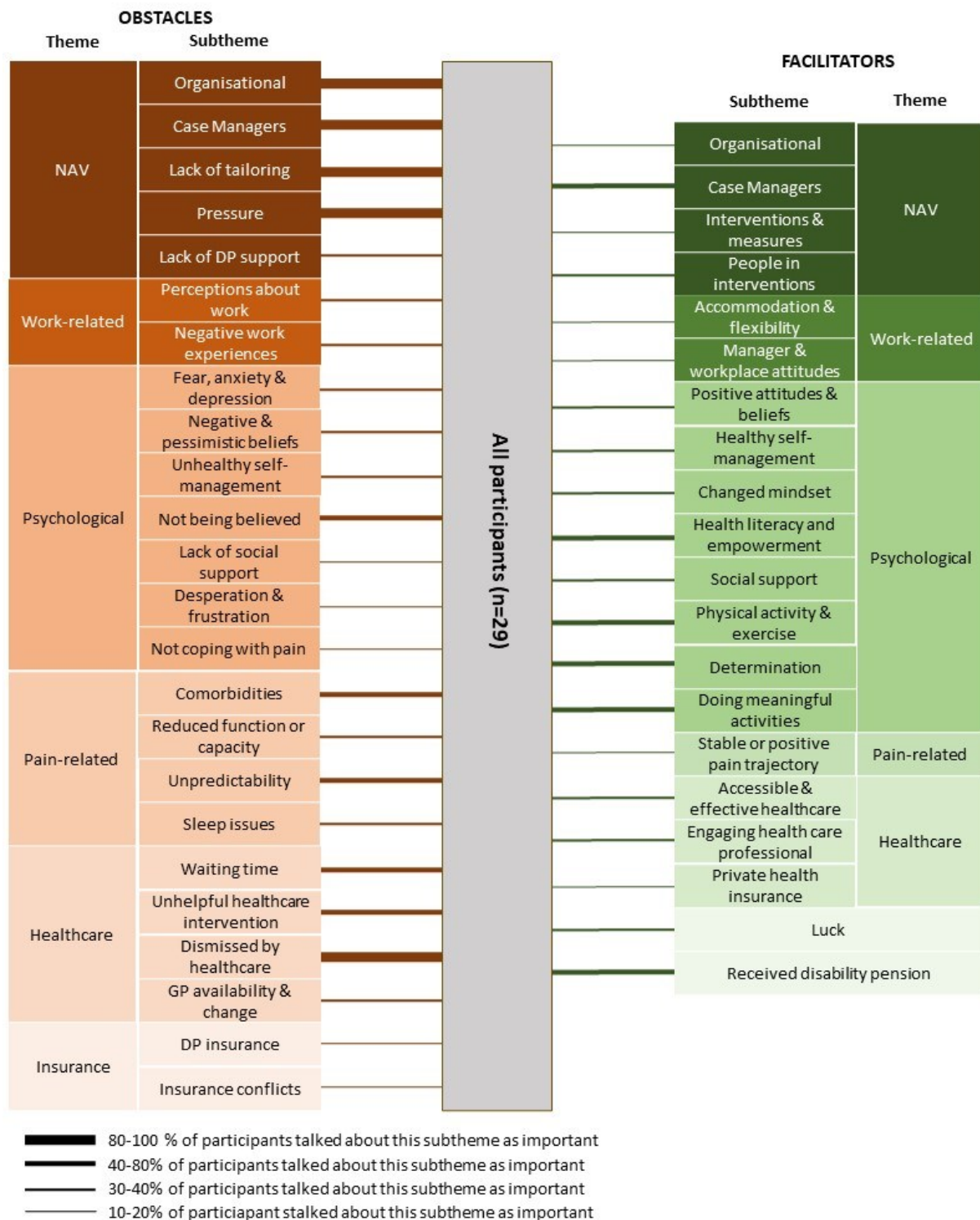

Supplement: Supplementary file 1 — Supplementary Material 1. [file 12889_2025_24747_MOESM1_ESM.pdf]
