# Supplementary material for: Obstacles and facilitators of return to work among people with persistent pain who receive benefit payments: an in-depth interview study
Source: BMC Public Health. 2025 Oct 21;25:3532. doi: 10.1186/s12889-025-24747-0 (PMC12539112; doi:10.1186/s12889-025-24747-0)
Supplement: Supplementary file 2 — Supplementary Material 2. [file 12889_2025_24747_MOESM2_ESM.pdf]

Supplementary File 1.

**Topic**	• **Questions (●) & prompts (○)**
**Welcome and introduction**	• Wish welcome, briefly explain the study and the interview process. Ask if they have any questions about the study or the information leaflet (information and consent obtained prior)<br>• What is the reason for you wanting to participate in this interview study?
**Demographics**	• How long have you had pain?<br>• How long have you been unemployed?<br>• Are you, or have you ever been on WAA/DP?<br>• How long on WAA (dates) and/or DP and what percentage?
**Persistent pain**	• Can you tell me how your pain developed and how it has affected your life?<br>○ Can you recall any key events during this time? (Related to pain)<br>○ How did that make you feel or think?<br>• Thinking about the future, do you think your pain will get better, worse or stay the same?<br>• Have you found anything that helps you to manage your pain / enables you to do more of the things you care about?
**Previous work**	• Have you worked previously? Could you tell me a little about what you did when you used to work?<br>• *During your time in work, before you stopped working due to your current health problem: did you encounter any health problems or pain? If so…*<br>○ *Did you have any time out of work (sick leave)?*<br>○ *How did you cope with it? What were the obstacles at the time and how did you manage to overcome them?*<br>• How did you eventually come to stop working?<br>○ Can you recall any key events during this time?<br>○ How did that make you feel or think?
**The 'Welfare system'**	• Can you share your experiences in dealing with government agencies (e.g., NAV) / case workers while managing your chronic pain and seeking employment support?<br>○ Can you recall any key events during this time?<br>○ How did that make you feel or think?<br>• Did your involvement with the welfare system present challenges or facilitators in your journey to return to work?<br>○ Can you recall any key events during this time?<br>○ How did that make you feel or think?
**Attempts of RTW**	• Can you share any thoughts and experiences on what you think hindered your return-to-work process?<br>○ Encourage them to discuss the nature of these challenges and how they attempted to overcome them.<br>• During an interview process (on application or in interview) or employment, did you have any reservation about disclosing/discussing your pain? [*Formulation depends on previous replies]*<br>• Explore any stigmatisation/discrimination/negative experiences and how this has influenced the return-to-work journey
	• Can you share any thoughts /experiences of what you think facilitated / might facilitate your return-to-work?<br>○ Encourage them to provide specific examples and elaborate on their thinking/experiences.<br>• Are there any specific factors or personal characteristics you think contributed to your return to work?
	• What do you think are / would be the most important obstacles or main challenges for not returning to work?<br>○ Encourage them to provide specific examples and elaborate on their thinking/experiences
**Personal characteristics 'me'**	• How will you describe your self-perceived strengths and weaknesses/limitations?<br>• How do you define your ability to bounce back from challenges and setbacks?<br>• How well have you been able to adapt to the limitations caused by chronic pain?<br>• What coping strategies have you found most effective in managing pain?<br>○ Discuss any specific skills they developed or enhanced during their unemployment period.
**Closing**	• Is there anything of importance that we have not covered or that you want to add?
