# Supplementary material for: Obstacles and facilitators of return to work among people with persistent pain who receive benefit payments: an in-depth interview study
Source: BMC Public Health. 2025 Oct 21;25:3532. doi: 10.1186/s12889-025-24747-0 (PMC12539112; doi:10.1186/s12889-025-24747-0)
Supplement: Supplementary file 3 — Supplementary Material 3. [file 12889_2025_24747_MOESM3_ESM.pdf]

Supplementary File 3.

Translated and the original Norwegian quotes are listed and those quotes that have been used in the article are italicised.

1. Obstacles

1.1 NAV-related obstacles

[366 quotes in total]

1.1.1 Organisation

[98 quotes]

Q1.1 *Because I felt like every time I got a NAV case manager, I had to go through the whole story again. They could come to a meeting without having looked at your file. So you had to start all over again, every time. And every time they had a new explanation about what we should do. Because then they come up with new ideas every time*

- *For jeg følte at hver gang jeg fikk NAV-rådgiver, så måtte jeg jo fram med hele historien. De kunne komme i møte uten å ha sett på journalen din. Så du måtte jo begynne helt på nytt igjen, hver gang. Og hver gang hadde de en ny forklaring om hva vi skal gjøre. For da kommer de med en ny ide hver gang.*

Q1.2 *NAV has a challenge when it comes to the terminology they use, the words they use for different things. It's not just academics who become sick or long-term ill.*

- *Der har jo NAV en utfordring i forhold til terminologien de bruker, hvilke ord de bruker på de forskjellige tingene. Det er jo ikke akademikere som bare blir syke eller langtidssyke.*

Q1.3 For the first four years on AAP, I was mostly on waiting lists. Waiting lists in the healthcare system, waiting lists at NAV, long processing times. You can't get hold of NAV; you can try calling NAV and see what happens. Then you end up with two hours in the phone queue.

- For de første fire årene på AAP så sto jeg mest på venteliste. Venteliste i helsevesenet, venteliste på NAV, lang saksbehandlingstid. Du får ikke tak i NAV, du kan jo prøve å ringe NAV og se hva som skjer. Da har du to timer i telefonkø.

Q1.4 I was told that there was an eight-month processing time

- Jeg fikk jo beskjed om at det var åtte måneders behandlingstid da

Q1.5 I also find it completely shocking that they change advisors so often to avoid forming a personal relationship with the user. I think it should be a huge advantage to know the user. That way, you know what kind of challenges this person has. And it also seems like they have too many users per advisor. Because they can't keep up. So that's why you have to be very proactive and constantly follow up. What's happening now? What are we doing now? Because if you don't do that, you hear nothing.

- det synes jeg også er helt sjokkerende at de skal bytte på så ofte for å ikke få et personlig forhold til brukeren. Det synes jeg burde være en kjempefordel at du

kjenner brukeren. Så du vet hva slags utfordringer denne personen har. Og det virker som også at de har alt for mange per rådgiver. For de klarer ikke å følge opp. Så det er derfor du selv må være veldig proaktiv og hele tiden mase. Hva skjer nå? Hva gjør vi nå? For hvis du ikke gjør det så hører du ingenting.

Q1.6 You never know who you'll encounter or what the person... The people you talk to at NAV have figured out that, okay, if I say yes to this, and when the call ends, it's no longer my responsibility. So when I ask the next person and mention what the previous one said, they respond, "No, that's not right, it's not like that." Then you're back to square one, and you have to start all over again.

- Du vet jo aldri hvem du treffer på, hva personen… de du snakker med på NAV, de har jo funnet ut at, ok, hvis jeg sier ja til dette, og når han legger på, så er det ikke mitt ansvar lenger. Så når jeg spør neste person, og sier det som den andre sier, ja, nei, men det stemmer jo ikke det, det er jo ikke sånt, ja. Så er de tilbake til starten igjen, og så må du begynne på nytt.

Q1.7 There has only been one general practitioner in the 23 years I have been unwell, so it is only one general practitioner who has collaborated with NAV and attended the meeting with NAV. We're talking about maybe five general practitioners over 23 years, so only one has attended the meeting with NAV. The other general practitioners have said that they do not have time, they do not have the possibility, maybe they do not bother. But I have actually only had one general practitioner who attended the meeting, shared some opinions, and was very cooperative. Otherwise, the cooperation between NAV and..., is very poor, and there is no communication between the general practitioner and NAV

- Det er bare en fastlege av de 23 årene jeg har vært dårlig, så det er kun en fastlege som har samarbeidet med NAV og vært med på møtet med NAV. Det er snakk om kanskje fem fastleger på 23 år, så det er kun en som har vært med på møtet med NAV. For da har fastlegene sagt at de ikke har tid, de har ikke mulighet, gidder kanskje ikke. Men jeg har faktisk bare hatt en fastlege som var med på møtet og sa noen sine meninger og var veldig samarbeidsvillig. Men ellers er det veldig dårlig samarbeid mellom NAV og…, og det er ikke kommunikasjon mellom fastlege og NAV.

Q1.8 No, I have not felt that there has been much [collaboration between NAV, the general practitioner, and others in the healthcare service]. Because the specialists and such that you visit, they send discharge summaries to the general practitioner. So it's actually the general practitioner who takes it up with NAV... but...

- ....Nei, det [samarbeid mellom NAV, fastlege og andre i helsetjenesten] har jeg ikke følt at det har vært noe særlig av. Fordi de spesialister og sånn du er hos, de sender epikriser til fastlegen. Så er det egentlig fastlegen som tar det med NAV.. men..

Q1.9 My general practitioner is not very fond of NAV. And that's kind of because... They are so inflexible, right? So it becomes like... I think the doctors feel a bit... They feel controlled by NAV. That it has to be done in this and that specific way

- Fastlegen min er jo ikke så veldig glad i NAV. Og det er litt sånn på grunn av... De er så lite fleksible da, ikke sant? At det blir sånn... Tror legene blir litt sånn... Føler seg styrt av NAV da. At det skal være på sånn og sånn og sånn måte.

1.1.2 Case managers

[44 quotes]

Q1.10 *Here it's all about figures and money and numbers. I don't know, it feels like that. You're just a number in line. You're not a person who's sitting there with health problems initially*

- *her er det tall og penger og nummer. Jeg vet ikke. Det føles sånn. Du er et nummer i rekka. Du er ikke en person som sitter der med i utgangspunktet helseproblemer.*

Q1.11 But at NAV, they do not inform you about what rights you have or what you can apply for to get help. Nothing. You have to figure out everything yourself. You have to sit and read everything on your own.

- men på NAV informerer meg ikke om hva slags rettigheter du har. Eller hva du kan søke på for å få hjelp. Ingenting. Du må finne ut av alt selg. Du må sitte og lese alt selv.

Q1.12 I couldn't just be myself... because then I felt like I was being suspected and labeled as lazy.

- Jeg kunne liksom ikke bare være meg selv da.. for da følte jeg meg mistenkeliggjort og stemplet som lat

Q1.13 Because they have no empathy or understanding of how you feel. They just follow rules, and that's how it is. When you're sitting there crying because they're sending you to yet another thing, it's just, well, too bad. If you don't participate, we'll stop your AAP. That's just the way it is.

- Fordi de har ingen empati eller forståelse på hvordan du har det. Fordi de skal bare følge regler og sånn og sånn er det. Når du sitter og gråter for at du blir sendt på en eller annen ting til, så er det bare, ja. Men hvis du ikke blir med på det, så stopper vi AAP-en din. Sånn er det bare.

Q1.14 Because this NAV case manager, even when you're lying there vomiting from pain, still wants to force you into testing and clarification. When, in the first place, you might deserve to be allowed to have a little peace at first

- Fordi at denne her NAV-veilederen, når du ligger og spyr av smerte, skal fortsatt tvinge deg på avklaring. Når du i utgangspunktet kanskje burde få lov å få litt ro i bunnen.

1.1.3 Lack of tailoring

[99 quotes]

Q1.15 *They group together people who struggle to get a job, because they don't know how to get a job, and those who are sick. And then they put everyone through the same process. I don't need to know how to write a resume or a cover letter, or, yeah... It was not matched with my needs… to get some guidance. But that type of help… there was no competence or access to it.*

- *De sammenstiller folk som sliter med å få jobb. Fordi de ikke vet hvordan de skal få jobb, og syke. Og så kjører de alle gjennom samme løpet. Jeg trenger ikke vite hvordan jeg skal skrive en CV eller en søknad, eller, ja. det ble en veldig liten match mellom mine behov. For å få veiledning. Det kunne faktisk vært noe jeg kunne trengt der også. Men den hjelpen… var ikke kompetanse eller tilgang på.*

Q1.16 And I also don't understand why everyone has to go through the same process when we are all different people with different diagnoses and challenges. They need to look more closely at what should be done for each person to help them get back to work. It cannot be the same for everyone.

- Og jeg forstår ikke heller at alle skal gjennom det samme når vi er alle forskjellige personer som har forskjellige diagnoser og utfordringer. Så må de se mer hva vi skal gjøre for den personen for å få den tilbake i arbeid. Det kan ikke være likt for alle.

Q1.17 Because I was on an immunosuppressive medication, and then they sent me to an elementary school, and the doctor was like, "hello...". So, it was like, and there was no alternative thinking regarding my education, or whether I could have done something...

- For da gikk jeg jo på en immunpressende medisin, og så sånn… sende meg på en barneskole, og så sånn, legen bare sånn, hallo.. Så det var på en måte, og det var heller ikke noe sånn, det var ingen som tenkte alternativt med tanke på utdannelsen min, eller om jeg kunne gjort noe…

Q1.18 And what has happened is that now everyone is forced into the workforce at any cost. And into any profession. Without taking health challenges into consideration.

- Og hva som har skjedd, det har jo skjedd at nå skal alle piskes ut i arbeidslivet koste hva det koste vil. Og i hvilket som helst yrke. Uten at det tas hensyn til helseutfordringer

Q1.19 The AAP expired. And then I said, what do we do now? I can't do anything else. So they say, 'we need to get you into work training.' And then I thought, what are they going to put me on now? Then he says, but do you have any suggestions? So I said, no, I don't have any. Then we start again. And then I get to work for such and such an amount of time and so many months. And then it starts all over again, the same mess. The same setup again.

- AAP-en gikk ut. Og så sier jeg, hva gjør vi nå da? Jeg kan ikke gjøre noe annet. Så sier de, "vi må ha deg på arbeidstrening." Og så tenkte jeg, hva skal de sette meg på nå da? Så sier han, men har du noen forslag? Så sier han, nei, jeg har ikke det. da begynner vi på'n igjen. Og så får jeg jobbe i så og så lang tid og så mange måneder. Og så blir det forfra i samme mølje. Samme opplegg igjen.

1.1.4 Distress related to NAV

[107 quotes]

Q1.20 *It's fear. I could be dreading a NAV meeting for a whole week in advance. It was absolutely terrible. I would go with heart palpitations and feeling nauseous. It's absolutely horrible. Migraine attacks and everything. Because your whole body tenses up. When you are constantly being threatened that they will take away your benefits, what do you do then? You are completely dependent on them. I found it incredibly tough.*

- *Det er frykt. Jeg kunne grue meg en uke før jeg skulle på NAV-møte. Det var helt forferdelig. Jeg gikk og hadde hjerteklapp og jeg var kvalm og. Det er helt forferdelig. Migrenanfall og alt mulig. Fordi du binder hele kroppen. For når du hele tiden blir truet med at de tar lønna di, hva gjør man da? Du er jo helt avhengig av dem. Jeg syntes det var innmari tøft.*

Q1.21 It's demanding in a way that drains a lot of energy to constantly deal with oneself, one's own future, one's own health. The risks you can get into, it's so scary to make a mistake. There's a lot of stress involved in constantly thinking about, what if I do something wrong now? What if they uncover something I didn't even know was a thing? What if I said something wrong? What if they don't believe me?

- Det er krevende på en sånn måte at det tapper veldig mye energi å forholde seg til seg selv hele tiden, sin egen fremtid, sin egen helse. De faresonene du kan gå inn på, det er så skummelt å trå feil. Det er veldig mye stress involvert i det, at du stadig vekk tenker på, hva om jeg gjør noe feil nå? Hva om de gjennomskuer et eller annet, som ikke jeg visste var en ting en gang? Hva om jeg har sagt noe feil? Hva om de ikke tror på meg?

Q1.22 Because you sort of transition from being a healthy person to a sick person, and then you have to, you've spent a lot of time processing the fact that you're actually so sick, and now you have to start defending that you're sick as well

- for du går opp på en måte fra en frisk person til en syk person, og så skal du på en måte, først har du på en måte brukt masse tid på å bearbeide at du faktisk er så syk, og så skal du begynne å forsvare at du er syk i tillegg.

Q1.23 If you don't do as NAV says, they can take your income. You can't say no. That's just how it is. And that's why I think it's completely wrong. If they had listened to the healthcare system back then and said she needs peace to get better, I think maybe I would have recovered sooner. Instead, I was held back

- Nei, hvis ikke du gjør som NAV sier, så tar de inntekta di. Du kan ikke si nei. Det er rett og slett det. Og derfor så tenker jeg at det blir jo helt feil. For hadde de hørt på helsevesenet den gangen og sagt ho må ha ro til å få bli frisk. Så tror jeg kanskje jeg hadde blitt friskere tidligere. I stedet så ble jeg holdt tilbake.

Q1.24 I think that with these invisible illnesses, we are not perceived as equally sick as if you were in a wheelchair or on crutches. We don't look sick. But every time I had a meeting with NAV, I would wear sweatpants and no makeup. I thought, then I actually look a bit sicker. And it's not to make myself out to be something I'm not or anything.

- Jeg tror veldig mye sånn som med de usynlige sykdommene, at vi blir ikke sett på som like syke på en måte enn om du hadde kommet i en rullestol eller på krykker. At vi ser jo ikke syke ut. Men hver gang jeg skulle på møte med NAV så gikk jeg i joggebukser og uten sminke og alt. Da tenkte jeg da ser jeg faktisk litt sykere ut. Og det er ikke for å gjøre meg til eller noen ting.

1.1.5 Lack of support for those on disability pension

[18 quotes]

Q1.25 *You could have thought that NAV could support you…or support me in getting back to work. But they say, no, you are completely disabled and cleared, and then that's true*

- *At de kunne gjennom tenkte at NAV kunne jo støtte deg i dag eller støttet meg til at jeg går i jobb. Men de sier jo det at nei, du er hundre prosent ufør og ferdig avklart, og da er det sant.*

Q1.26 *When you become a disability pensioner, that's the end of it. The only thing I have now is my regular doctor. And all he can do for me is prescribe painkillers*

- *Når du blir ufør, så blir du ferdig med det. Det eneste jeg har nå er fastlegen min. Og alt han kan gjøre for meg og skrive meg smertestillende.*

Q1.27 And then there's this issue with payment adjustments, disability benefits while working. If you've been following the newspapers, you know that many people are getting repayment demands. You can go in and try to figure it out yourself. It's completely hopeless. And when you are sick and have to use your energy to try to re-enter the workforce and work as much as you can because you don't want to be excluded from the workforce. No one wants that.

- Også dette her med avregning, uføretrygd i arbeid. Som du har fulgt litt med i avisen og alle som får tilbake betalingskravet og sånt. Du kan gå inn og prøve å finne ut av deg selv. Det er helt håpløst. Og når du da er syk, skal bruke krefter på prøver og kommer tilbake i arbeidslivet og jobber det du kan fordi du har ikke lyst å ramle ut for arbeidslivet. Det er det ingen som har lyst til.

1.2 Work-related obstacles

[47 quotes in total]

1.2.1 Perceptions about work

[18 quotes]

Q1.28 *And I feel a bit like that, that if I had found the perfect job that could adapt, right? And then with the option of working from home and having flexible hours, right? It doesn't work like that.*

- *Og det føler jeg litt på, at hadde jeg funnet en perfekt jobb med som kunne tilpasse seg, ikke sant? Og da med hjemmekontor og ha muligheten fleksitid, ikke sant? Det funker ikke sånn.*

Q1.29 Who is it, in a way, that would want to hire someone, like you say at 10%, but where you, in a way, come and go as you please

- Hvem er det som på en måte vil ansette noen, sånn som du sier 10%, da, men hvor du på en måte sånn kommer og går litt sånn som du vil.

Q1.30 And it's very understandable given the job market, but the thing is, if you, say, begin working and you're scheduled to work on Tuesdays and Thursdays, and then you're not well on a Tuesday. So you call in and say you're sick, right? No job can put up with that, and it's completely understandable, isn't it?

- Og det er jo veldig forståelig i forhold til jobbmarkedet, men det er jo det at hvis du på en måte, hvis jeg nå skulle begynne å jobbe, du jobber tirsdag og torsdag, ikke sant? Og så er jeg dårlig den tirsdagen da. Så ringer jeg inn og så er jeg dårlig, ikke

sant? Det er ingen jobb som orker å forholde seg til det, og det er fullt forståelig, ikke sant?

Q1.31 It’s not so easy for employers to arrange accommodations and make it work. Yes, some would prefer to have everything fit within the standard working hours. That’s essentially the biggest problem.

- Det er ikke så lett for arbeidsgivere å på en måte tilrettelegge og få det til. Ja, da vil noen gjerne ha det mest mulig innen den arbeidstiden som er. Det er på en måte det som er det største problemet.

1.2.2 Previous negative experiences from work

[29 quotes]

Q1.32 *Because I heard that the others have to work more to make up for you. Because you're not here. And that's not nice to hear.”*

- *Fordi jeg fikk jo høre at de andre må jo jobbe mer for å ta igjen for ditt. Fordi du ikke er her. Og det er ikke noe hyggelig å høre.*

Q1.33 If I don't manage to work 50%, then we don't need her. Period. We don’t need someone who works 20% and has been on sick leave so much and is perceived as unreliable and all that.

- Hvis jeg ikke kommer å jobbe 50% så har vi ikke brukt for hun. Ferdig. Vi trenger ikke noen som jobber 20% og så mye sykemeldt som hun har vært og upålitelig og sånt.

Q1.34 I submitted medical certificates from my regular doctor because, you know, after car accidents or something like that, it just hasn't helped, plain and simple. It was some kind of concussion, and I couldn't sit at the computer for very long, and I got tired very quickly. And now, I haven't received any accommodations or understanding from my employer. Even though I’ve provided the documentation.

- Jeg leverte legeeklæringer fra mitt faste leger, fordi, nå, etter bilulykker eller noe sånt, det har ikke hjulpet rett og slett. Fordi, på en måte, det var noen sånn hjernerystelse, og da kunne jeg ikke sitte ved pc veldig lenge, og jeg var veldig sliten fort. Og nå, jeg har ikke fått noen tilpasninger eller forståelse fra arbeidsgiveren. Selv om jeg har levert det.

Q1.35 I started to understand that they weren't investing in me. So in a way, they gave me tasks that maybe could be delivered in three, four, or five months. So if I don't complete them, it doesn't really matter. In that sense, I began to feel less included in all the work processes.

- jeg begynte å forstå at de ikke satset på meg. Så på en måte at de gir meg oppgaver som kanskje nå kan levere som tre eller fire, fem måneder. Slik at hvis jeg ikke gjør det, så gjør det ikke noe. Så på en måte at jeg begynte å føle meg mindre inkludert i alle arbeidsprossesene.

Q1.36 And with my last employer, we initially agreed that I would work two days a week, but everything got turned upside down. Then he basically wanted me to work five days a week. So it just became more and more, and in the end, I had to just throw in the towel. They didn't take into account that I needed handicapped parking and such.

- Og som siste arbeidsgiveren, da avtalte vi egentlig at jeg skulle jobbe to dager i uken, men alt ble snudd om. Altså ville han egentlig at jeg skulle jobbe fem dager i uken, da.

Så det ble bare mer og mer og mer, og til slutt så måtte jeg bare kaste inn håndkleet. De tok ikke hensyn til at jeg trengte handikapparkering, og sånn.

1.3 Psychological

[164 quotes in total]

1.3.1 Fear, anxiety, depression

[28 quotes]

Q1.37 *I think it's very easy when you get sick yourself, to become scared. Pain makes you scared. And then it's very easy to retreat into that bubble and feel sorry for yourself.*

- *jeg tror det er veldig lett når du blir syk selv, så blir du redd. Smerter gjør at du blir redd. Og da er det veldig lett å gå inn i den boblen, og sitte og synes synd i seg selv.*

Q1.38 Because after the first year of AAP, I was really down. I thought, I can't handle this. I was really depressed.

- For jeg hadde jo, etter det første året i AAP, så var jo jeg langt nede. Jeg tenkte at jeg, dette orker jeg ikke. Så jeg var skikkelig deprimert var jeg

Q1.39 ...but naturally, I was scared to begin with. Because I had heard so many horror stories and negative things. I was especially worried about my diagnosis and thought, will they understand it? Will they realize that this isn't just a broken bone? There was a lot of fear involved.

- men naturligvis var jeg redd til å begynne med. Fordi jeg hadde hørt så mange skrekkhistorier, og negative ting. Jeg var spesielt bekymret for diagnosen min, og tenkte at kommer de til å forstå det? Til å skjønne at dette ikke bare er et brekt bein. Det var mye frykt inni bildet.

Q1.40 Because my pain and such are greatly affected by how stressed I am and the overall uncertainty. So, I developed the reason I initially went to DPS [District Psychiatric Center] just because I was depressed.

- Fordi mine smerter og sånt kommer veldig av hvor stressa jeg er, og hvor mye generell usikkerhet og sånn. Så jeg utviklet egentlig grunnen til at jeg dro på DPS i starten bare fordi jeg var deprimert

Q1.41 I think, in a way, it also became a sort of traumatizing experience for me, that when I needed a bit more understanding or at least patience, I didn't receive it. And actually... Now, in a way, I'm scared to have a job because of those relationships, because I can't work physically, and with these relationships, I see it's difficult now. Because I have anxiety, and I don't want to go through the same thing again.

- Jeg tror på en måte det også ble på en måte traumatiserende erfaring for meg, at når jeg på en måte trengte litt mer forståelse eller i hvert fall tålmodighet, og det har jeg ikke fått, og faktisk... Nå, på en måte, jeg er skremt til å ha jobb på grunn av de relasjonene, fordi jeg kan ikke jobbe fysisk, og med relasjoner, jeg ser at det er vanskelig nå. Fordi jeg har angst, og jeg vil ikke oppleve det samme mer.

1.3.2 Negative and pessimistic beliefs

[27 quotes]

Q1.42 From the lower part of my back. It looks quite bad, like a poor 90-year-old's bad back. It looks terrible.

- Fra nedre deler rygg. Så den ser ganske stygg ut, som en dårlig 90 år gammel dårlig rygg. Den ser elendig ut.

Q1.43 If it had only been one thing, maybe I could have found a solution. But since there are so many things, I see that it becomes nearly impossible. That's probably what is leading me to end up being 100% disabled now.

- Hadde det bare vært en ting, så kanskje jeg hadde funnet noe. Men siden det er så mange ting, så ser jeg at det blir nesten umulig. Det er nok det som gjør at jeg ender opp med å bli 100% uføre nå.

Q1.44 So I believe my ailments stem from that, and I think that my diagnosis, if I were to have one, is fibromyalgia. That makes the most sense to me

- så jeg tror mine plager kommer av det, og jeg tror at min diagnose, hvis jeg skulle hatt en, er fibromyalgi. Det er det som gir mest mening for meg.

Q1.45 When I was completely debilitated, when I couldn't even hold a glass of water, and it went on for months, I thought that I was ruined for life.

- når jeg var helt utslått, når jeg ikke kunne holde et glass vann, og det gikk måneder; Jeg tenkte at jeg var ødelagt for livet.

1.3.3 Unhealthy self-management

[38 quotes]

Q1.46 *But what happens is that I push myself too far because I find it so enjoyable. And then I start to feel pain, but I just ignore it, and eventually, I can hardly stand on my feet.*

- *Men det som skjer er at jeg strekker meg for langt, for jeg synes det er så gøy. Og så begynner jeg å kjenne smerter, og så bare overhører jeg det, og så klarer jeg nesten ikke å stå på beina til slutt.*

Q1.47 That was the only thing I wanted, so I think I pushed myself a lot, trying to get better as quickly as possible. And then it probably stagnated a bit because of that stress, maybe.

- Det var det eneste jeg ville så jeg tror jeg har stressa meg veldig opp da, for at jeg skulle bli bedre igjen så fort som mulig. Og så har det nok stagnert litt på grunn av det stresset da kanskje

Q1.48 And then I'm a bit skeptical again, but deep down, I'm really just hoping and waiting for it to go away, so I can get back to work, right? That's what I'm hoping for.

- Og da er jeg litt mer sånn skeptisk igjen, men jeg sitter jo egentlig og håper og venter på at det blir borte, også er jeg tilbake på jobb, ikke sant? Det er jo det jeg håper på.

Q1.49 So, it's probably quite a lot of medication then. Yes, it is. I originally thought that the more I took, the less inflammation there would be in my body and the better I would feel. But it turns out that I've likely been taking way too much for a long time. And that has affected my mind and everything else.

- Så det er nok ganske mye medisiner da. Ja, det er det.Jeg trodde egentlig at jo mer jeg tok, det var jo mindre betennelse i kroppen og jo bedre skulle jeg være. Så viser det seg at jeg har sikkert tatt alt for mye lenge. Og det har gjort at det går ut over hodet og sånn.

1.3.4 Not being believed

[27 quotes]

Q1.50 *And then there's this thing with society... like with taking walks. You can manage to go for a walk, but you can't manage to go to work. You can travel on vacation, but you can't manage to travel to work. You feel like you have to constantly defend yourself just to live.*

- *Og så er det litt det her med at samfunnet er... litt dette her med å gå tur. Du klarer å gå tur, men du klarer ikke å gå på jobb. Du klarer å reise på ferie, men du klarer ikke å reise på jobb. Du føler at du må forsvare deg hele tiden for at du skal leve*

Q1.51 *But there are many, doctors who have told me, both at the hospital and other doctors have said to me, that the pain is psychological.*

- *Men det er jo mange som, leger som har sagt til meg da, både på sjukehuset og andre leger som har sagt til meg det, at det er psykisk at du har vondt.*

Q1.52 Because it is stigmatization, plain and simple. Because those who struggle with things you can't see are looked down upon and judged. They are judged by other healthy people. And it's not because those people are evil, it's because of ignorance

- Fordi det er stigmatisering, enkelt og greit. Fordi de som sliter med ting som du ikke kan se, de blir sett ned på og dømt. De blir dømt av andre friske mennesker. Og det er ikke fordi de er onde, det er på grunn av uvitenhet.

Q1.53 But I said that I wished I had cancer. I don't actually wish for that, but I had a very, very strong need to prove that it was... and it wasn't just with NAV, but with everyone around me.

- Men jeg sa at jeg skulle ønske jeg hadde kreft. Jeg gjør jo ikke det, men jeg hadde veldig, veldig behov for å liksom bevise at det var… og det var ikke bare med NAV, men liksom hele omgivelsene.

Q1.54 It’s so negative in a way. But because it’s not visible—if I were walking with a crutch, or if I were in a wheelchair, then maybe they would see it... When it’s not visible, it’s like people might think you just don’t want to work. I’m so scared that people will start to look at me differently. I don’t want that. I think it creates so much negativity. It's probably foolish. Many people say you just have to be open about it, but I don’t feel comfortable with that. For my mental well-being, it’s much better to pretend everything is normal.

- Det er jo så negativt på en måte. Men på grunn av at det ikke er synlig, hvis jeg hadde gått med krykke, eller hvis jeg hadde gått i rullestol, så hadde de kanskje sett det... Når det ikke er synlig, så er det sånn at du kanskje ikke vil jobbe. Jeg er så redd at folk skal begynne å se på meg på en annen måte. Jeg vil ikke. Jeg synes det skaper så mye

negativitet. Det er sikkert dumt. Det er jo mange som tenker at du må bare si det, men jeg føler meg ikke komfortabel med det. For min psykisk er det mye bedre å late som alt er normalt.

1.3.5 Lack of social support

[10 quotes]

Q1.55 Otherwise, I've heard from those around me... they say things like, "I think there's always something weird going on with you," right, because I've been through these potential hypothesis diagnoses and shared that story. And then it changes. So in the end, I didn't want to say anything more, because I could hear myself that it sounded crazy.

- Ellers så har jeg jo hørt fra omgivelsene… så er det litt mer sånn, «synes du feiler så mye rart jeg», ikke sant, for jeg har jo vært igjennom disse potensielle hypotese diagnosen og så har man hatt den fortellingen. Og så forandrer det seg. Så til slutt så hadde jeg jo ikke lyst å si noe mer, for jeg hørte jo selv at det ble kokko

Q1.56 I don't really have anyone I can ask for money or anything. If something goes really wrong, I've always had to manage on my own.

- jeg har liksom ingen jeg kan spørre om penger og sånn. Hvis det er et eller annet ille ute, så har jeg alltid liksom måtte klare meg på egenhånd.

Q1.57 It's constant nagging from NAV, family, friends, and everyone. It's constant nagging like, "When are you going back to work? Are you back at work? Are you working? Are you back at work again?"

- Det er et evig mas ifra, både NAV, familie og venner og alt sånn. Det er evig mas liksom, ja, når er du på jobb igjen? Er du på jobb igjen? Er du på jobb? Er du på jobb igjen?

1.3.6 Suicidal ideation

[7 quotes]

Q1.58 *I have been very down at times, so... so far down that I tried to end it all. I've been down there many times. And that's because the pain has become so intense and I've faced so much adversity both with NAV and the healthcare system*

- *Jeg har vært veldig nede i perioder, så… så langt at jeg har både prøvd og gjort det slutt på livet for å si det sånn. Der nede har jeg vært mange ganger. Og det er på grunn av at smerten har blitt så stor og at jeg har jo møtt så mye motgang både hos NAV og hos helsevesenet.*

Q1.59 There's a lot of sorrow, it's very exhausting to be in pain every day. Sometimes I just want to jump out of the window to end it. Medication does not help much. The feeling that you don't want to live comes back quite often.

- Det er mye sorg, det er veldig slitt som du har det vondt hver dag. Noen ganger har jeg bare lyst å hoppe ut av vinduet så det skal ta slutt. Medisin hjelper ikke noe særlig. Det følelsen av at du ikke har lyst til å leve, den kommer ganske ofte tilbake.

Q1.60 I think that at the time I thought it was the worst, that there was nothing... I might as well give up. It wouldn't lead anywhere.

- Jeg tror nok at da tenkte jeg at det var det verste at det er ikke noe... kan like gjerne gi meg. Det vil jo ikke gå noen vei.

1.3.7 Desperation and frustration

[13 qoutes]

Q1.61 And then you're sitting there, saying to yourself, "Well, after eight months, I have to do something to get better, right?" So you end up just throwing money out the window on everything, or something like that. You become desperate.

- og så sitter du sånn, ja, men da, åtte måneder, jeg må gjøre noe ja, for å bli frisk, ikke sant, så du blir jo bare, du kaster jo penger ut av vinduet etter alt, eller noe sånt, man blir jo desperat

Q1.62 It was very frustrating. Why can't I sleep? Why do I wake up 30 times during the night? Why am I so exhausted? For me, it helps to have an explanation. And when I don't have an explanation, that's when the frustration comes. I need to find a reason.

- da var det veldig frustrerende. Hvorfor klarer jeg ikke å sove? Hvorfor våkner jeg 30 ganger om nettene? Hvorfor blir jeg så sliten? For meg hjelper det å ha en forklaring. Og når jeg ikke har en forklaring, det er da frustrasjonen kommer. Jeg må finne ut en grunn.

Q1.63 No, it's a terribly difficult situation. You don't live, you just exist. Not only that, but you are simply fighting to get through it. It's not good to feel like this all the time, you end up feeling hopeless.

- Nei, det er en del forferdelig vanskelig situasjon. Man lever ikke, man eksisterer. Ikke bare selv, men kjemper rett og slett. Det er ikke noe godt å ha det hele tiden, det blir jo oppgitt.

1.3.8 Not coping with the pain

[8 quotes]

Q1.64 Most of the days are centered around the illness. You wake up with it, and it’s the first thing you feel the moment you wake up. You feel that you are sick, and in a way, it's the last thing you feel when you go to bed at night. It affects a large part of your day.

a. det er jo mesteparten av dagene det dreier seg om sykdommen da. Du våkner med det og kjenner det med første du våkner, du kjenner når du våkner at du er sjuk , og på en måte det er det siste du kjenner når du legger deg i sengen på kvelden med da, så det påvirker en ganske mye av dagen da.

Q1.65 It's not that it's a pain I have to scream from. It's not that intense, but it's there all the time. You cant catch a break, right? You’re getting crazy

b. Det er ikke sånn at det er en smert jeg må skrike av. Det er ikke så sterk, men det er jo hele tiden. Du får ikke pause, ikke sant? Du holder på å bli gal.

1.4 Pain-related obstacles

[97 quotes]

1.4.1 Comorbidities

[23 quotes]

Q1.66 *And eventually the fatigue, because it was really the fatigue that became the biggest problem over time. I think the pain… in the worst case, I can live with it. But being fatigued, that's, that's torture. I think it's worse than being in pain.*

- *Og etter hvert utmattelsene, for det var egentlig utmattelsene som ble det største problemet etter hvert. Jeg synes smerten, det klarer jeg i verste fall å leve med. Men det å være utmattet, det er jo en, det er jo tortur. Jeg synes det er verre enn å ha vondt.*

Q1.67 It's pain, and eventually, it affects the whole body. I suddenly get a lot of infections—throat infections, urinary tract infections...

- Det er smerter, og så blir det da, går det til slutt utover hele kroppen. Jeg får plutselig masse infeksjoner, halsbetennelser, urinvegsinfeksjoner..

Q1.68 I have many diagnoses, both mental and physical. They have just piled up over time. I hate diagnoses, but I have them from head to toe, mentally as well. So it has affected me a lot.

- Jeg har veldig mange diagnoser, psykisk og fysisk. Det har bare ballet på seg. Jeg hater diagnoser, men jeg har det fra topp til tå og psykiske også. Så det har påvirket meg veldig.

Q1.69 In addition to that, I also have a so-called IBD—Crohn's disease, a severe intestinal condition that has, at times, put me out of action quite a bit.

- I tillegg til det så har jeg en så kalt IBD-chronstarm altså en alvorlig tarmsykdom som i perioder har satt meg ganske ut.

Q1.70 Because I have fatigue, it really kicks in quickly when I get tired. Then I get headaches and I also have tinnitus with some hearing loss that I've had since the injury.

- Fordi jeg har fått fatigue, og den slår det egentlig veldig kjapt til når jeg blir sliten, da blir det hodeverk og så har jeg tinnitus med litt nedsatt hørsel har jeg fått etter skaden.

1.4.2 Reduced function / capacity

[31 quotes]

Q1.71 *When I, for example, explain that I can spend an entire day to a whole week getting housework done, or when I talk about all the things I can't manage… or that I can go to work, but when I come home I am completely exhausted for the rest of the day*

- *Når jeg for eksempel forteller at jeg kan bruke en hel dag til en hel uke på å få gjort husarbeid, eller at jeg forteller om alle de tingene jeg ikke får til, eller at jeg klarer å gå på jobb, men når jeg kommer hjem så ligger jeg flatt ut hele dagen*

Q1.72 Yes, it is really about being in so much pain, and when the arm was so painful, I became numb and heavy. But it's very much about the energy drain from being in pain, and losing concentration, not finding words, not feeling present.

- Ja, det er egentlig det med det å være så smertepåvirket, og når da denne armene var så vond, så ble jeg sånn nummen og tung. Men veldig det der med å, altså det energitapet med å gå og ha vondt, og det å miste konsentrasjon, ikke finne ord, ikke føle at en er tilstede.

Q1.73 No, from what I've seen, it's not getting better. It just keeps getting worse every year. It's a bit scary.

- Nei, sånn som jeg har sett nå så blir det jo ikke bedre. Det blir hele verre. For hvert år som går. Det er litt skummelt.

1.4.3 Unpredictability

[31 quotes]

Q1.74 *I can't find any causal relationships that make it so… if A then B. It's not that once I figure something out, then I can just do this, and it will work. It has a life of its own*

- *jeg klarer ikke å finne noen årsakssammenhenger som gjør at hvis A så B. Det er ikke sånn at med en gang jeg har funnet ut at ja, da kan jeg liksom gjøre sånn, så stemmer ikke det. Det lever sitt eget liv.*

Q1.75 But I think it is very difficult, because I can see for myself, I know that I have the capacity to work. The problem is just that my physical state is extremely unpredictable

- Men jeg synes at det er veldig vanskelig, for jeg ser jo selv, jeg vet at jeg har kapasitet til å jobbe. Problemet er bare at formen min er helt ekstremt uforutsigbar

Q1.76 And it's like my energy levels vary so much, so some days I function really well, and then the next day it's completely the opposite. And I don't understand what others understand, it's difficult to predict how and why.

- Også er det sånn at jeg varierer jeg meg så i energinivå, så noen dager funker jeg kjempebra, og så neste dag er det helt sånn motsatt. Og jeg skjønner ikke det andre skjønner, det er vanskelig å forutse hvordan og hvorfor.

Q1.77 But I can also get such pains occasionally. Then my partner says that we need to go to the emergency room. Because suddenly I can't walk on one leg. It hurts so much that I had to go and get crutches from the garage. Because I couldn't put weight on my right leg. And the next day it's gone.

- Men jeg kan også få sånne smerter som innimellom. Så sier samboeren min at nå må vi dra ned på legevakten. For plutselig kan jeg ikke gå på det ene beinet. Det gjør så vondt at jeg måtte opp og hente krykker i garasjen. Fordi jeg klarte ikke å tråkke ned på høyre ben. Og dagen etterpå er det borte.

1.4.4 Sleep issues

[14 quotes]

Q1.78 *Because I struggle a lot with sleep. Because I don't... I'm in so much pain. That you... You can't fall asleep, and then you wake up again because it hurts. And then you are completely foggy in the morning*

- *For jeg sliter mye med søvn. For at jeg ikke har... Har så vondt i kroppen. At du.. Sovner ikke, også våkner du igjen av at det gjør vondt. Og så er du helt tåkete om morgenen.*

Q1.79 But the nights can be quite... because you struggle to sleep. And if you then struggle to sleep, it affects the next day, and then it just snowballs.

- Men nettene kan jo bli ganske… fordi du sliter med å få sove. Og hvis du da sliter å få sove, så går det ut over neste dag, og så baller det seg på.

Q1.80 So I can wake up in the middle of the night and feel like I'm freezing to death. Even though I'm covered with a winter duvet, I lie there trembling all over. And then when I finally get warm, I get so hot that I have to take off the duvet. It's something with the temperature regulation that my body can't manage. And then it affects both the quality of sleep. It's a combination of exhaustion, lack of sleep, and pain, everything all together in a way. It just becomes too much somehow.

- Så jeg kan våkne mitt på natta og holde på å fryse ihjel. Selv om jeg ligger med vinterdyne så ligger jeg og skjelver over hele kroppen. Også når jeg først blir varm så blir jeg så varm at jeg må ta dyna. Det er noe med temperaturen som kroppen ikke klarer å regulere. Og da går det liksom både på det at du sover dårlig. Det er liksom både utmattelsen og lite søvn, smerter, alt i alt på en måte. Så bare ble det for mye på en måte.

1.5 Healthcare-related obstacles

[126 quotes]

1.5.1 Waiting

[19 quotes]

Q1.81 *What breaks most people is the waiting time... that's what it is.*

- *Det som tar knekken på de fleste er ventetiden.. det er det.*

Q1.82 *Firstly, it's the healthcare system. It just takes such a long time before you get... it took a good six months before I saw a specialist.*

- *Altså, for det første er jo det der helsevesenet. Som det tar jo da så lang tid, før du får... det tok et godt halvår til jeg kom til spesialist.*

Q1.83 I understand the reasoning that the Conservative Party was trying to apply pressure, NAV was supposed to speed up the process a bit, but it doesn't work, there's a one-year waiting time at hospitals, right, it doesn't align.

- Jeg skjønner tankegangen at Høyre skulle legge press på, NAV skulle liksom få litt fortgang i den prosessen, men det funker jo ikke, det er jo et års ventetid på sykehus, ikke sant, det snakker jo ikke sammen.

Q1.84 Because you can imagine, there's a three-month waiting period just to get in... So let's say I wait three months to see a rheumatologist and then a neurologist

- For du kan tenke deg, det er jo tre måneders ventetid bare for å komme inn på... Så si jeg at jeg venter tre måneder på å komme til en revmatolog og så nevrolog.

1.5.2 Unhelpful healthcare utilisation

[60 quotes]

Q1.85 *But I felt like they threw a lot of pills at me. That was unfortunate. Especially since I was so young. I was only about 20. I got fifty morphine tablets a week. It was quite a lot. It was hard to quit.*

- *Men jeg kjente at det ble kastet ganske mye piller på meg. Det var dumt. Særlig siden jeg var så ung. Jeg var vel bare 20. Jeg fikk sånn femti morfintabletter i uka. Det var ganske mye. Det var vanskelig å slutte med.*

Q1.86 *I have tried different therapists. I have tried different therapies. I go for a while, three, four, five months. No improvement. In most of the therapies I started, the focus was only on the pain. I give up.*

- *Ja, også har jeg prøvd forskjellige terapeuter. Jeg har prøvd forskjellige terapier. Jeg går i en stund, tre, fire, fem måneder. Ingen forbedring. I de fleste terapiene som jeg begynte, ble det bare fokusert på smerte. Jeg gir opp*

Q1.87 They never managed to decide where this belonged. Which department at the hospital it belonged to. I had to go back to my GP to get a new referral to the hospital…There were three different ones. I went to specialists and all sorts of things. I have experienced that so many times.

- De fikk aldri bestemt seg hvor dette hørte hjemme. Hvilken avdeling på sykehuset det hørte hjemme. Jeg måtte tilbake til fastlegen for å få ny henvisning til sykehuset.. Det var tre forskjellige. Jeg gikk til spesialister og alt mulig. Det har jeg opplevd så mange ganger.

Q1.88 Yes, I have gone private and paid a lot, a lot of money for physiotherapists, chiropractors, and manual therapists and everything. To somehow see if it helps. But it helps a little at the moment, and then it's back again.

- Ja, jeg har gått privat og betalt masse, masse penger i fysio, kiropraktor og manuellterapeuter og alt. For å så på en måte se om det hjelper. Men det hjelper litt akkurat da og så er det tilbake igjen

Q1.89 I have a slight feeling that when I had an emergency surgery in (city), they made a mistake. Because they really like to do emergency surgeries and only do things halfway, if you see what I mean. They try to fix it, but it still doesn't turn out as they hope. It happens all the time.

- jeg har en liten følelse av det at når jeg ble hasteoperert i (by), at de gjorde en feil. For at de er veldig glad i å hasteoperere og gjøre ting delvis halvveis, hvis du ser hva

jeg mener. At de prøver å fikse det, men at det allikevell ikke blir sånn som de håper. Det skjer støtt og stadig.

1.5.3 Dismissed by healthcare

[39 quotes]

Q1.90 *Even when I've gone to the emergency room and cried, they've referred me to an urgent appointment. And at the urgent appointment, I've been clearly told that they can't help me because I don't have psychosis. So, in a way, I have to be extremely down to get help from the DPS or other institutions offering mental health support.*

- *Selv om jeg har kommet på legevakt, og jeg har grått, de har henvist meg til akutte time. Og på akutte time har jeg fått klart beskjed at de ikke kan hjelpe meg, fordi jeg ikke har psykose. Så på en måte, jeg må være ekstremt nede for å få hjelp fra DPS eller andre institusjoner med psykisk hjelp.*

Q1.91 *He thought that my problems were psychological, and I was like, no, but you become very mentally worn out from being in pain all the time, being tired all the time, and not having money.*

- *han mente da at problemene mine var psykisk, og så ble jeg sånn, nei, men man blir veldig psykisk nedbrutt av at man på en måte har vondt hele tiden, er sliten hele tiden, og ikke har penger.*

Q1.92 *The worst are those who work in the healthcare system. I've heard doctors say, "No, but you see, here we deal with life and death." Or... "Well, well, we have to learn to live with the little things." Comments like that, but it's mostly the healthcare system. Because then everything accumulated—the disbelief, no hope, nothing that could be done, and it was all about how you handle it. Now you just have to smile and be happy, and it'll be fine. And that wasn't a solution.*

- *De verste er de som jobber i helsevesenet. Jeg har jo hørt leger som sier, «nei men du skjønner hos oss så jobber vi med liv og død». Eller… «ja ja, litt småtteri må vi lære oss å leve med.» Sånne, men det er stort sett helsevesenet. For da kumulerte alt det jeg ikke ble trodd på, ikke noe håp, ingenting å få gjort, og det er bare måten du tar det på. Nå må du bare smile og være glad så blir det bra. Og det var jo ikke en løsning.*

Q1.93 *When it comes to diffuse issues, you're kind of cast aside in the healthcare system. Because not all pain can be explained.*

- *Når det er diffuse ting, så er du en sånn parikast i helsevesenet. For det er ikke all smerte som lar seg forklare.*

Q1.94 *The general practitioner, you know, they're just like I said about NAV, they're overwhelmed with work, right, so everything is in and out, in and out, so it kind of falls on you then.*

- *Fastlegen, ikke sant, de har jo, de sitter jo akkurat sånn som jeg sa om NAV, de er jo dynget ned i arbeid, ikke sant, så alt blir sånn inn ut, inn ut, så da blir det litt opp til deg selv da,*

Q1.95 I switched general practitioners a few years ago because I felt I was getting poor help from the previous one. She just ran tests and said, 'No, there's nothing here

- Jeg byttet fastlege for noen år siden før jeg syntes jeg fikk litt dårlig hjelp av den fastlegen. For hun tok bare prøver og sa “nei her var det ingenting”

Q1.96 So then I was just told that they couldn't do anything for me, they couldn't help me. So I was discharged from Rikshospitalet and sent back to him in [PLACE]

- Så da fikk jeg bare beskjed om det at vi ikke kan gjøre noe for deg, vi kan ikke hjelpe deg. Så da ble jeg avsluttet på Rikshospitalet og sendt tilbake til han i STED.

Q1.97 Yes, but I have been rejected by the (rehabilitation center). I can't move forward. But then they can't move forward either. It's a bit like, what do you do then? Because NAV can only send you to work-oriented rehabilitation. They can't send you to (special rehabilitation centers) or those that aim to improve quality of life in some way. Because if NAV requires you to go to that, then they should also be able to send you to it. You shouldn't have to go through your general practitioner. And then maybe a poor medical certificate causes you to be rejected. Because then you can't move forward with your assessment through NAV.

- Ja, men jeg har fått avslag av (rehabiliterings-senter). Jeg kommer ikke videre. Men da kommer ikke de videre heller. Det er litt sånn, hva gjør man da? Fordi nav kan bare sende til arbeidsrettet rehabilitering. De kan ikke sende til (spesial-rehabiliteringssentre) eller sånne som går på å få bedre livskvalitet på en måte. For hvis NAV krever at du skal på det, så må de også kunne sende deg på det. Du skal ikke da gå gjennom fastlege. Og at da kanskje en dårlig legeerklæring gjør at du får avslag. Fordi da kommer du ikke videre med utredningen din gjennom NAV

Q1.98 The doctors in there can't figure anything out. I was referred further, but they can't find anything either. And I'm really wondering what's actually happening, because no one knows

- Legene der inne klarer ikke å finne noe ut av det. Jeg ble henvist videre, de finner ikke noe ut av det. Og jeg lurer jo fælt på hva som faktisk skjer, for det er det ingen som vet.

1.5.4 Change in GPs

[6 quotes]

Q1.99 Then there were several temporary doctors, and I had to explain everything again to each one of them. I think everyone has different experiences. It depends on who you have as your doctors, whether you suddenly lose your general practitioner, or if you need a new one. I've probably dealt with 20 doctors during that period, without it being my fault that the system keeps changing.

- Også ble det flere vikarer og som målforklare på nytt til bare eneste av dem. Jeg tror alle har forskjellige opplevelser. Hvem du har som leger, om du plutselig mister fastlegen, om du må ha ny. Jeg har sikkert hatt med 20 leger å gjøre i den perioden. Uten at det har vært min skyld at systemet bare bytter.

Q1.100 With my last doctor, I've had eight general practitioners throughout this process as well, right?

- Med den siste legen min, jeg har jo hatt åtte fastlegen gjennom dette løpet også, ikke sant?

1.6 Other

[10 quotes in total]

1.6.1 Insurance for disability

[6 quotes]

Q1.101 *From the first earned krone [NOK], in my case, I lose my insurance money, which is [NOK] 5000 kroner a month, plus the child supplement for my disability, which is [NOK] 4700 kroner. So that means I lose [NOK] 10,000 kroner. If I start working again, no matter the percentage, I lose it. And what I mean by that is, of course, I want to work, but that one day could cost me so much.*

- *Fra første tjente kroner, for min del, så mister jeg forsikringspengene mine, som er 5000 kroner i måneden, pluss barnetillegget på uføren min, som er 4700 kroner. Så det vil si at jeg mister 10 000 kroner jeg. Hvis jeg begynner å jobbe igjen, samme hvor mye prosent, så mister jeg den. Og det jeg mener med det da, er det at selvfølgelig, jeg vil jo jobbe, men den ene dagen kan koste meg såpass mye*

Q1.102 So I also have insurance, and I receive a payout each month from (insurance company). Because I've never been able to get that kind of insurance myself, as I've always been sick. So I've never managed to get insurance on my own. Since my employer provided it for me. So I receive almost [NOK] 6000 per month from them in addition. And that helps when you're only getting 66 percent of your salary. It really does help.

- Så jeg har jo også forsikring, så jeg får utbetalt hver måned fra (forsikringsselskap). Fordi jeg har aldri klart å få en sånn type forsikring selv, for jeg har alltid vært syk. Så jeg har aldri klart å få en forsikring selv. Så siden arbeidsgiver hadde den på meg. Så jeg får utbetalt nesten 6000 i måneden fra dem i tillegg. Og da hjelper det på når du bare får 66 prosent av lønna din. Det hjelper jo det.

1.6.2 Insurance conflicts

[4 quotes]

Sitater:

Q1.103 *Well… I can say that I have had my battles with the insurance company, because it has been an insurance matter.*

- *vel jeg kan jo si det at jeg har hatt mine kamper med forsikringsselskap, for det har vært en forsikringssak.*

Q1.104 I've had a case with patient injury compensation, and I've been rejected twice, where I presented my case, and they believe that it has been handled correctly.

- Jeg har hatt sak med pasientskadeerstatning, og jeg har fått avslag to ganger, hvor jeg har lagt fram saken min, og de mener det at det har vært behandlet riktig.

2. Facilitators

2.1 NAV

[98 quotes in total]

2.1.1 Organisational

[11 quotes]

Q2.1 My experience is at least that NAV works much better if it is clear and obvious what is wrong with you

- min erfaring er i hvert fall at NAV fungerer mye bedre hvis det er klart og tydelig hva som feiler deg.

Q2.2 There has been very little turnover in NAV. At least here. It's mostly the same people. I haven't interacted with many people there, really. So, it's definitely an advantage that they know you and... Yes, they know a bit of your history

- Det har vært veldig lite utskifting i NAV. Iallfall her da. Det er stort sett de samme. Det er ikke mange jeg har vært innom egentlig da. Så det er helt sikkert en fordel med at de kjenner deg og... Ja, vet historien litt da.

Q2.3 The job I have now was offered to me through NAV, because they have my CV

- Den jobben jeg har nå ble jeg tilbudt gjennom NAV, for de har CV-en min.

2.1.2 Case managers

[52 quotes]

Q2.4 *Then she said, "Now you will get one year, where you can get what you need." And I was startled. "If you want additional education, further training, whatever. So that you can return to work". When I left her office, I cried. And I told her, "If you see someone jumping and dancing down the street, that's going to be me." Because I got what I needed, and I was back at work a year later.*

- *Og så sa hun at nå skal du få et år på deg hvor du kan få det du vil ha. Og jeg er skvatt. hvis du vil ha noe videreutdanning, ekstrautdanning, hva som helst. Sånn at du kan komme tilbake i jobb. da jeg gikk ut fra kontoret derfra, så grein jeg. Og jeg sa til henne, hvis du ser noen som hopper og danser nedover der, så er det meg. For da fikk jeg det jeg trengte å var tilbake på jobb året etter.*

Q2.5 I just remember that she, before I met her, and that it was kind of a collaboration, how are we going to get things in place, and how are we going to, like. She was also very, very skilled, and she wasn't too focused on following the rules, I think. She used her own judgment.

- jeg bare husker at hun også, før jeg møtte meg, og at det på en måte var et samarbeid, hvordan skal vi få ting på plass, og hvordan skal vi, liksom. Hun var også veldig, veldig dyktig, hun var heller ikke så opptatt av å følge reglene, tror jeg. Hun tenkte selv.

Q2.6 Because she said that you are way too young to apply for disability benefits. You will get... we will sort this out, like. So she managed to get me into retraining. Because she was very keen on helping. Yes, so that was great.

- For hun sa det, at du er altfor ung til å søke ufør. Du skal få... dette skal vi ordne, liksom. Så hun fikk meg inn på at jeg fikk omskolering. For hun var veldig på at det var hun ville hjelpe. Ja, så det var kjempebra.

Q2.7 They have told me, the NAV case manager, that you are not functioning, this is not working, and getting insight into what they write and say, and being part of some of the processes, being empowered, has been very helpful. Being able to see, for example, here is the work capability assessment I have written about you

- At de har sagt til meg, NAV-veileder, at du fungerer ikke, dette går ikke, og at det å få innsyn i det de skriver og sier, og være med på en del av den prosessen, bli myndiggjort, det har vært veldig hjelpsomt. Det å kunne se for eksempel, her er arbeidsevnevurderingen jeg har skrevet av deg,

2.1.3 Measures and intervention

[20 quotes]

Q2.8 *If I hadn't received the education I did, I might not have had that opportunity. I might have had to work less, perhaps being partially on sick leave or something similar. So, I am very grateful that NAV helped me get on the path of retraining.*

- *Hadde jeg ikke fått den utdanning jeg har fått, så hadde jeg kanskje ikke hatt den sjansen. Da hadde jeg kanskje måttet jobbe mindre, at jeg hadde vært delvis sykemeldt eller sånn. Så jeg er veldig glad for at NAV har hjulpet meg inn på sporet av omskolering*

Q2.9 because I was doing an internship at [company] in January to test my work capability. And since I enjoyed it so much there and really want to achieve 100 percent, they consider it as a confirmed work capability. It's the best job I could have had

- for jeg var på praksis på [*bedrift*] i januar, for å teste arbeidsevnen. Og siden jeg trivdes så godt der, og egentlig har veldig lyst til å få til 100 prosent, så anser de det som ferdig avklart med arbeidsemnet. Det er den beste jobben jeg kunne hatt

Q2.10 Being assigned a job specialist, I didn't find that very easy to accept. I would have preferred to handle it on my own and try to find something. But it is indeed a good opportunity to get help and connections in many places, and it's actually great for getting in touch with people and getting responses

- Det der å få utdelt en jobbspesialist, det synes jeg nok ikke var så greit. Jeg ville helst ha ordnet dette på egenhånd og prøve å finne noe. Men det er jo en god mulighet til å få hjelp og kontakt om mange plasser og er jo kjempegod egentlig til å få tag i folk og få svar.

2.1.4 People in measures and interventions

[17 quotes]

Q2.11 *I got the feeling that she genuinely wanted to help me and shielded me from things that would harm rather than help me. So, it was very... and I was completely turned around because I went in with such negative experiences that had pretty much shaped me. So, I was*

very pleasantly surprised, and she has been the one who has helped me move forward. And it is because of her that I am where I am today.

- *Fikk følelsen at hun hadde riktig lyst til å hjelpe meg, og skjermet meg for ting som heller skader meg enn hjelper meg. Så det var veldig… og jeg ble snudd helt, for jeg gikk jo inn med så negativ erfaring som jeg bar ganske preg av. Så det ble veldig positivt overrasket, og hun har jo vært den som har hjulpet meg videre. Og hun gjør jeg er der jeg er i dag.*

Q2.12 and then I have had fantastic advisors and employers, and the whole support system around me has basically been playing on the same team

- og så har jeg hatt et fantastisk veiledere og arbeidsgivere, og hele støttappet rundt meg har jeg liksom spilt på samme lag da.

Q2.13 Then I got to try a new internship through [company]. And the woman at [company], she was absolutely amazing. It was... maybe... I mean, without her, I wouldn't be where I am now

- Så fikk jeg prøve ny praksis igjennom [*bedrift*]. Og hun i [*bedrift*], hun var helt fantastisk. Det var... Det er kanskje... Altså uten hun hadde jeg ikke vært der nå.

2.2 Work-related facilitators

[43 quotes in total]

2.2.1 Acoomodation and flexibility

[27 quotes]

Q2.14 *So, both the work tasks and the work environment… meaning that I had a centered roller mouse and a proper chair and a sit-stand desk, and all this, right, that things were adapted, and you had the opportunity to take a break and go for a walk, or sit down on another chair, or something like that. When it was accommodated, it worked well.*

- *Altså, både arbeidsoppgaver, men arbeidssituasjonen, altså at jeg hadde sentrert rullemus og skikkelig stol og hev- og senkpult, og alt dette sant at ting ble tilrettelagt, og du hadde muligheten til å ta deg en pause og gå deg en runde, eller sitte deg ned på en annen stol, eller noe sånt. Når det var tilrettelagt, så gikk det fint*

Q2.15 And I also think that the fact that I received the retraining is significant, so now I have a job where I have the possibility to adapt. Not all jobs allow for that. But this one actually does.

- Og jeg tenker også at det faktisk er at jeg har fått den omskoleringen, så jeg har en jobb hvor jeg har mulighet å tilpasse. Det er jo ikke alle jobber som tillater det. Men denne gjør faktisk det.

Q2.16 So, I have received some accommodations there with certain tasks I don't do, so that I don't overuse my muscles, because we have seen that it's the best way for me to manage this. I hope that this can be a way for me to get out a bit more.

- Så jeg har fått littrettelegging der med at enkelte ting jeg ikke gjør, sånn at jeg ikke bruker musklene, for jeg har sett at det er mest mulig at jeg skal klare det her da. Så jeg håper at det kan være en måte å få komme meg ut litt.

2.2.2 Manager and workplace attitudes

[16 quotes]

Q2.17 *We have many very interesting discussions that I can't have with anyone else, in a way, because no one else understands them. And then there's… being received well. From the first day, I was included in a way, and I think that means a lot*.

- *Vi har veldig mange interessante diskusjoner som jeg ikke kan ha med noen andre på en måte, for ingen andre som forstår de. Og så er det å bli tatt godt imot. Fra første dag så ble jeg inkludert på en måte, det tror jeg har veldig mye å si*

Q2.18 At the interview, I shared everything. I did that because I felt that there was a good dialogue here. And I've felt that all along as well. They have their flaws and shortcomings. But there's such a good culture. Otherwise, I wouldn't have wanted to work there either.

- Så på intervjuet fortalte jeg alt. Det gjorde jeg fordi at jeg følte at her er det en god dialog. Og det har jeg følt hele veien også. De har sine feil og mangler. Det er en sånn god kultur. Ellers hadde jeg ikke ville jobbe der heller.

Q2.19 So I just informed them that I could skip that one, and it was no problem at all. They are very adaptable there, and they even tell me to take a break now and then. And then I sit down for a bit, and they tell me to notify them if it becomes too much, and just say no. So they are really like that [gives thumbs up].

- Så ga jeg bare beskjed om at jeg kunne slippe akkurat den, og det var ingen problem. Så der er det veldig sånn at det har vært tilpasning, og med at de sier at nå må du ta en liten pause. Og så setter jeg meg ned litt også, gi beskjed, hvis det blir for mye, og bare si nei. Så de er veldig sånne liksom.

Q2.20 The boss, she asks how much I can manage to work. And she respects when I say no. She makes adjustments accordingly. Then she asks if I'm okay and if I need to do something else that's easier. So she's really nice.

- Hun sjefen, hun spør om hvor mye jeg klarer å jobbe. Og så tar hun nei for nei. Og da tilpasset hun jo. Og så spurte hun, går det bra? Trenger du å gjøre noe annet som er enklere? Så hun er jo veldig grei da

2.3 Psychological

[307 quotes in total]

2.3.1 Positive attitudes and beliefs

[34 quotes]

Q2.21 *I think it must be that I am generally quite positive. Instead of seeing problems, I mostly see solutions. It doesn’t occur to me to give up in a way, because things always work out.*

- *Det tror jeg må være at jeg stort sett er ganske positiv. I stedet for å se problemer så ser jeg stort sett løsninger. Det faller meg ikke inn å gi opp på en måte, for det ordner seg alltid.*

Q2.22 I can cry and feel really down and go quite low, but then afterwards, I don't know. I do get some strength back. But I try to tell myself, 'it will get better.

- Jeg kan jo grine og stå veldig sånn og gå ganske langt ned, men så etterpå så vet jeg ikke. Jeg får jo litt styrke igjen. Men jeg prøver å "det kommer til å løsne seg."

Q2.23 Get up and say that today is going to be a good day, have a set routine, that's very important

- Stå opp og si at i dag blir det en god dag, ha fast rutiner, det er veldig viktig.

Q2.24 So I keep finding more and more of these kinds of things, and along with diet and exercise and such, I'm very hopeful. I probably will never get completely rid of it, but it can definitely be managed

- Så jeg finner liksom flere og flere sånne typer ting, og litt med kosthold og trening og sånn, men jeg er veldig håpefull, jeg tror jeg blir sikkert aldri helt kvitt det, men det kan jo absolutt styres.

2.3.2 Healthy self-management

[34 quotes]

Q2.25 It's hard to explain, but it's also about thinking oneself healthy in a way. It sounds really silly, but I believe we have a bit more, how shall I say, I need to phrase this correctly. When you think that today is bad and today is like this, I think it actually becomes that way.

- Det er vanskelig å forklare, men det også tenke seg frisk på en måte. Det høres helt teit ut, men jeg tror vi har litt mer, at man, hva skal jeg si, må ordlegge meg riktig. At når man tenker at i dag er det dårlig og i dag er det sånn, så tror jeg at det blir sånn.

Q2.26 And of course, you look at role models and see that if you sleep well, exercise, and get out in the fresh air, all those things contribute to making things better

- Og selvfølgelig så ser man på modeller og ser at hvis du sover godt, og du trener, og du kommer de ut i frisk luft, altså alle de tingene som skal være med på at ting er bedre da

Q2.27 I understand that it's painful; I've been there. But it doesn't get better by just... And it doesn't help to say that it won't work. You have to try first.

- Jeg forstår at det er vondt, jeg har vært der. Men det blir ikke det bedre av det, hvis du bare... Og det blir ikke bedre å si at det kommer ikke til å fungere. Du må prøve først.

2.3.3 Changed mindset

[30 quotes]

Q2.28 *So I used to be a career woman. It's quite a big step from doing that to working in a [type of job], but that's where I need to be now.*

- *Så jeg var jo en sånn karrieredame. Så det er et ganske stort steg fra å gjøre det til å være i en [type jobb], men det er der jeg skal være nå.*

Q2.29 You learn from experience that you get much further if you accept the situation, but it is a process. You can't just decide that it's something you do. There's something, I don't know what it is, that can unlock it, leading to acceptance.

- Man har lært fra tidligere at jeg kommer mye lenger hvis man aksepterer situasjonen, men det er jo en prosess. Så man kan jo ikke bare bestemme seg for at det er noe man gjør. Det er noe med å, jeg vet ikke hva det er, som kan løsne det, at det fører til å aksept.

Q2.30 The thing with accepting... but what I've noticed is that accepting the situation has helped me

- Akkurat det å akseptere… men det jeg merker er det å akseptere situasjonen, at det har hjulpet meg.

Q2.31 It has changed. What I've found to be very important for me, when it comes to pain and managing pain, is my mantra: 'it's not dangerous, it's just painful

- det har endret seg. Det jeg kjenner har vært veldig viktig for meg, når det gjelder smerter og håndtering av smerter. Det mantraet jeg har, at det er ikke farlig, det er bare vondt.

2.3.4 Health literacy og empowerment

[29 quotes]

Q2.32 *I think it's a bit about not being so scared anymore. "If it feels like this, it might hurt. Is this a good idea? Am I doing this correct now? Will it get worse? Is this smart? I can handle this. My body is strong, it can handle this. Everything is good, everything is fine. If it gets worse, I'll take it easier next time. Relax a bit more. There's nothing structurally wrong with my body." And then I remind myself of that. That I am healthy, I am strong, I am safe, and it's going to be okay. That has probably had some impact on feeling stronger and approaching exercises in a slightly different way.*

- *jeg tror det handler litt om at jeg ikke blir så redd lenger for alt. Hvis det gjør sånn, så blir det sikkert vondt. Er dette noe lurt? Holder jeg på med dette nå? Kommer dette til å bli verre? Er det smart? Dette tåler jeg. Kroppen min er sterk, den tåler dette. Alt er good, alt er greit. Hvis det blir verre, så tar jeg det litt roligere neste gang. Slapper litt mer av. Det strukturelle i kroppen er egentlig helt fint. Og da minner jeg meg på det da. At jeg er frisk og jeg er sterk og jeg er trygg og det går bra. Det har sikkert satt noe innvirkning på at en føler seg sterkere og går inn i treninger for en litt annen måte.*

Q2.33 But getting the diagnosis and reading a bit about it, and meeting a rheumatologist who is an expert on the condition, and understanding, quite simply, that there are errors in the pain signals, but it's not dangerous. That acceptance has been very important.

- Men å få den diagnosen og lese på litt om det, og møte en revmatolog som er ekspert på tilstanden, og forstå rett og slett at det er feil i smertesignalene, men det er ikke farlig. Den akseptansen har vært veldig viktig.

Q2.34 I think that knowledge has been the key for me. Gaining a deeper understanding of how pain issues work.

- Jeg tenker at kunnskap har vært det som har vært nøkkelen for meg. Det å få en dypere innsikt i hvordan smerteproblematikk fungerer.

Q2.35 So my strength is probably that I can read, understand what I read, and use that knowledge to benefit myself.

- Så styrken min er nok at jeg kan lese, og forstå hva jeg leser, og bruke den kunnskapen til å gjøre litt nytte for meg.

2.3.5 Social support

[30 quotes]

Q2.36 Also, I think I'm lucky to have a good network. Additionally, I've been fortunate to receive a good education. I believe that helps. And I have the ability to... network, among other things, right? So I've been able to get in and know people.

- Også tror jeg at jeg er heldig og har et godt nettverk. Også har jeg vært heldig å ha en god utdannelse. Tror det hjelper. Og jeg har evnen til å… nettverke blant annet, ikke sant? Sånn at jeg har kommet meg inn og kjent folk.

Q2.37 I am married and have a very supportive husband. And I can talk to my sister and friends.

- jeg er jo gift og har en veldig støttende mann. Og søster og venninner kan jeg snakke med.

Q2.38 And then my husband has said, 'This isn't working.' And I think without the support I've had from my husband, I wouldn't be where I am today. He has been absolutely amazing.

- også mannen som har sagt, “dette går ikke.” Og jeg tror uten den støtten, jeg har hatt av mannen altså, så hadde jeg ikke vært der jeg er i dag. Så han har vært helt fantastisk altså.

Q2.39 That thought came quite early, and then it was my family and my close friends who supported me or were there for me, not for themselves. So luckily, my social network was very large. I still have many people around me.

- Den tanken kom ganske tidlig, og da var det familien min og de nærmere venninne mine som bidrog for meg, eller som var der for meg og ikke for seg selv. Så heldigvis så var det sosiale nettverket mitt veldig stort. Jeg har fortsatt veldig mange rundt meg.

2.3.6 Physical activity and exercise

[34 quotes]

Q2.40 *...so I go and exercise, no matter how bad I feel; it's almost like I always go and exercise. And when I say exercise, it's not like how I used to say it before. It's just about getting out there; it's for the sake of my mental health.*

- *så går jeg rett og trener, uansett hvor dårlig jeg er, så er det nesten sånn at jeg alltid går og trener da, og da, når jeg sier trening, så er det ikke sånn som jeg hadde sagt før, da er det, det er bare å komme seg ut, det er for psykens skyld.*

Q2.41 It's a bit easier to handle when I keep myself busy

- Det er litt sånn lettere å håndtere når jeg holder meg i gang.

Q2.42 I function best when I'm out walking. So I've started taking very long and frequent walks.

- Jeg fungerer best når jeg er ute og går. Så har jeg begynt å gå veldig lenge og mye turer.

2.3.7 Determination

[62 quotes]

Q2.43 *I think that when you've been at home for a while, and you don't have any money, you don't have the energy, you don't have the desire, you're just in pain and exhausted and losing hope, taking steps towards a goal is so important.*

- *Og jeg tror at når man har gått hjemme en stund, og man har ikke økonomi, man har ikke ork, man har ikke lyst, man har bare vondt, og er sliten, og mister håpet, at da liksom ta de skrittene mot et mål, tror jeg er så viktig.*

Q2.44 From day one, it was always me who had to say I wanted to do something, even to get a company health service doctor. It was me who made the contact.

- For det var jo fra dag 1, det var alltid meg som måtte si at jeg ville gjøre noe, til og med for å få bedriftshelsetjenestelege. Det var meg som tok kontakten.

Q2.45 It was me who had to come up with a suggestion for where I could try a work placement.

- Det var meg som måtte komme med et forslag hvor jeg kunne ta en arbeidsutprøving.

Q2.46 So I've set myself a goal, and when I achieve that goal, it feels good. Setting goals is important to me; it helps me overcome the pain in a way.

- så har jeg satt meg et mål, så har jeg i hvert fall klart det målet, og å sette seg en mål, det synes jeg er viktig, så det virker for meg da, å sette meg et mål, med å så klare, og så vinne over smerten på en måte.

Q2.47 And it's precisely that determination to stay in shape and be fit that makes my body function despite everything.

- Og det er nettopp den viljen da til å holde meg i form til å være sprek som gjør at kroppen tross alt virker.

Q2.48 I haven't wanted to go on disability, so I've done everything I can to avoid it.

- Jeg har ikke hatt lyst til å bli ufør, så jeg har gjort alt for å slippe unna det.

2.3.8 Doing meaningful activities

[24 quotes]

Q2.49 *Like volunteer work. Trying to be a bit engaged and thinking that I have to somehow live with my limitations. But I believe that shouldn't stop me from doing everything. So, I think it's partly that I don't want to just stay at home.*

- *Liksom frivillig arbeid. Prøvd liksom å være litt engasjert og tenke det at jeg må på en måte leve med at jeg kan mine begrensninger. Men jeg mener at det skal ikke stoppe meg i alt for det. Så jeg tror det er litt det at jeg har ikke lyst å bare være hjemme.*

Q2.50 And I think that my drive has kept me occupied all the time. I've always had something to do. Whether it was studying or, when I was job hunting, volunteering. That has been so important.

- Også tenker jeg at jeg har hatt med et visst driv at jeg har holdt meg opptatt hele tiden. Jeg har alltid hatt noe å gjøre. Enten studier, og når jeg har vært arbeidssøkende så har jeg jobbet frivillig. Det har vært så viktig.

Q2.51 But for example, I worked as a volunteer at a community center, helping in the kitchen by baking cakes and preparing some food. Just feeling important and needed, and knowing that what you do matters.

- Men for eksempel jobbet frivillig, og da har jeg vært på frivillig sentral og stått på kjøkkenet og baket kaker og laget litt mat. Og bare det å kjenne seg viktig og behøvd, og at det man gjør betyr noe.

Q2.52 I can drive you if you need it, like taking you to the doctor. It means a lot to me to be able to do that. I don't expect anything in return. It's just nice to be able to help someone.

- Jeg kan jo kjøre deg, hvis du trenger det, så gjør jeg det. Eller kjører deg til legen. Det gir meg veldig mye å kunne gjøre det. Jeg skal ikke ha noe tilbake. Det er bare hyggelig at jeg kan hjelpe noen.

2.4 Pain-related facilitators

[16 quotes in total]

2.4.1 Stable or positive pain trajectory

[16 quotes]

Q2.53 *And since I still have pain, there is some form of nerve pain related to it. Because my pain levels have become much more stable after I had surgery.*

- *Og i og med at jeg fortsatt har smerter, så er det en eller annen form for nervesmerter i forbindelse med det. Fordi jeg har jo blitt mye mer stabil i smertetilstanden min etter at jeg ble operert.*

Q2.54 "Now I'm much better, and that's one of the reasons I got assigned a job specialist last fall. I've been feeling much better since the summer, actually

- nå er jeg mye bedre, så det er jo en av grunnene til at jeg har den jobbspesialisten som ble koblet på i høst. Så er jeg mye bedre egentlig fra i sommer, så har jeg vært bedre.

2.5 Healthcare-related facilitators

[61 quotes in total]

2.5.1 Accessible and effective healthcare

[25 quotes]

Q2.55 *And then I was referred to the pain clinic in [anon]. It was only then that I truly got proper help. That was really when I felt like, okay, now I am getting proper help.*

- *Og da ble jeg jo henvist til smertepoliklinikken i [skjult]. Og da var det først da jeg fikk skikkelig hjelp. Det var jo virkelig da jeg på en måte følte at ok, nå får jeg skikkelig hjelp.*

Q2.56 *I attended a rehabilitation program at [anon] two years ago, and it was a very positive experience because everyone was in the same... How should I put it? Everyone was in the same boat. Everyone had their own burdens. We didn't talk about problems and illnesses and such. There was a reason we were there, whether we saw it or not. It was a very positive experience to be among like-minded individuals, even though we were all different.*

- *Jeg var jo på en sånn rehabilitering på [skjult] her for to år siden, og det var en veldig fin opplevelse, for der var alle i samme... Hva skal jeg si? Alle var i samme båt. Alle hadde sine laster. Vi snakket ikke om problemer og sjukdommer og sånt. Det var en grunn for at vi var der, om vi så det eller ikke. Det var en veldig fin opplevelse å være blant likesinnede, selv om vi var jo forskjellige alle sammen.*

Q2.57 I also underwent a very good treatment with a woman who was an osteopath. She taught me a lot about tension and relaxation, and how to feel the difference. I received some excellent relaxation exercises from her.

- Jeg var også på en veldig god behandling, hvor det var en dame som var osteopat, og hun lærte meg mye om det med spenning og avslapping, og det å kjenne på forskjellen. Og jeg fikk gode øvelser som var avslapningsøvelser

Q2.58 For example, I have participated in group therapy, and it has actually helped me to see the bigger picture. When people share their experiences, you understand that you are not alone in it.

- For eksempel, jeg har vært på gruppebehandling, og faktisk det har hjulpet meg å se litt større på bildet. Fordi når folk deler sine erfaringer, du forstår at du ikke er alene i det.

Q2.59 Much of it is also due to cognitive therapy. It's about working on oneself, spending a lot of time and focusing on oneself to figure things out. Yes, talk therapy, a lot of that.

- Mye på grunn av helt også kognitiv terapi. Litt sånn det å jobbe med seg selv. Bruker mye tid og fokus på seg selv og å finne ut av ting. Ja, samtaleterapi, mye sånt.

2.5.2 Engagig healthcare professionals

[30 quotes]

Q2.60 *I have a very good doctor who is very understanding and very good when it comes to pain. He has competence regarding pain. So, he and I have agreed that the most important thing is that I have a quality of life. To stabilize the pain so that I have a quality of life, that I am able to work a little, go out for walks. Basically, to be able to feel good.*

- *Men nå har jo jeg en veldig flink lege som er veldig forståelsesfull og veldig flink når det gjelder smerte. Og har en del kompetanse på smerte da. Så jeg og han har jo blitt enige om at det viktigste er at jeg har en livskvalitet. Få smerten stabile sånn at de har en livskvalitet, at jeg klarer å være litt i jobb, klarer å være litt ute og gå tur. Klarer å ha det bra på en måte.*

Q2.61 So I've had her for a year and a half now. I've had a good... well, good conversations with her. She has been willing to do the work, to read my story a bit and... Or had the time to do the work.

- Så hun har jeg hatt nå i halvannet år. Hun har jeg hatt et godt… altså en god samtale med. Hun har jo orket å gjøre jobben, om å lese seg selv litt tilbake og... Eller ha tid til å gjøre jobben.

Q2.62 You should get out and work, because that's the best thing, even if it's just a little. I feel that he is a bit younger, a bit more... Yes, I don't know. It's more like 'give it a try.' See if I can handle it. It's the best thing, even if you can only work a little.

- Du bør, komme deg ut i jobb, for det er det beste uansett om det bare er litt. Jeg føler at han er litt mer ung, er litt mer. Ja, jeg vet ikke. Litt sånn at prøv, prøv heller ut. Se om det jeg kan ta det. Det er det beste uansett om du klarer å jobbe litt.

2.5.3 Private health insurance

[5 quotes]

Q2.63 *What has helped me is that my employers had insurance for me. That way, I can get an appointment with a neurologist or rheumatologist within three days. Even when I needed surgery, I was told that they would schedule the operation, and I could get an appointment in a week.*

- *det som har hjulpet meg er at arbeidsgiverne har hatt forsikring på meg. Sånn at jeg kan få time etter tre dager hos nevrolog eller revmatolog. Til og med når jeg skal operere, så fikk jeg beskjed om at vi tar operasjon. Kan få time om en uke.*

Q2.64 But I am incredibly grateful that I have had insurance. It is the best investment. You do get to see specialists, but with treatment insurance, you get there faster. So, in a way, it's an unfair system. I got my surgery quickly, and everything has moved very fast for me. Because of treatment insurance.

- Men jeg er utrolig glad for at jeg har forsikringer hatt. Det er den beste investeringen. Du kommer jo til de spesialistene, men ved behandlingsforsikring kommer du fortere. Så egentlig er det et urettferdig system. Jeg var rask på operasjon, og alt har gått veldig fort hos meg. På grunn av behandlingsforsikring.

2.6 Luck

[17 quotes]

Q2.65 *Then I got to try a new work-placement through [anon]. And the woman at [anon], she was absolutely fantastic. It was... It's perhaps... I mean, without her, I wouldn't be where I am now"*

- *Så fikk jeg prøve ny praksis igjennom Falk. Og hun i Falk, hun var helt fantastisk. Det var... Det er kanskje... Altså uten hun hadde jeg ikke vært der nå.*

Q2.66 *Well, it's NAV (Norwegian Labour and Welfare Administration) that pays for these... But I was lucky this time to... met someone... and sometimes you are lucky... She was someone who managed to... that she maybe had enough clout to dare to... not just put me through the grinder, because they need to check boxes and write reports to NAV*

- *det er jo NAV som betaler disse... Men jeg var heldig denne gangen at.. traff på en.. og noen ganger er man jo heldig.. Hun var på en måte en som klarte å... at hun kanskje hadde litt pondus nok til å tørre å.. ikke liksom kjøre meg igjennom den her NAV kverna, for de skal tikke av å skrive rapport til NAV.*

Q2.67 But then, along the way, I have had a really great advisor at NAV. I have been very lucky.

- Men så har jeg hatt underveis veldig fin veileder på NAV. Jeg har vært veldig heldig.

Q2.68 Then I attended some sort of assessment courses, and we took a lot of tests for all sorts of things there, and they actually suggested that I could pursue further education. So that was really fortunate, actually

- Da var jeg på noen sånn avklaringskurs og så tok vi jo masse test for alt mulig der og så foreslo de egentlig der at jeg kunne ta en videreutdanning. Så det var jo kjempeflaks egentlig.

Q2.69 It depends on who you meet there; it's better to meet someone who stands their ground, who can help you, who can fight for you, in a way.

- Det spørs hvem du møter der, det er bedre å møte den som står på sitt, som kan hjelpe deg, som kan krige for deg på en måte.

2.7 Recieved disability benefit

[28 quotes]

Q2.70 *So he was very much about the fact that you gain a bit of surplus which you are in control of. You don't have NAV over you. You don't have anyone who is going to test you out. So that's something that can help. I've tried to take that to heart, that now it's basically just me. And now I control myself. And in a way, what I want, nobody is coming to monitor me. I am not on any measures. So I think that helps.*

- *Så han var veldig på at du får litt overskudd som det er du som styrer. Du har ikke noe NAV over deg. Du har ikke noen som skal prøve deg ut. Så kan det være å hjelpe. Så jeg har prøvd å ta det til meg at nå er det på en måte bare meg selv. Og nå styrer jeg selv. Og på en måte, hva jeg vil, det er ikke noen som kommer og kontrollerer meg. Jeg er ikke på tiltak. Så jeg tror det hjelper.*

Q2.71 *And perhaps get myself some jobs eventually, and build that up. One advantage of disability benefits is that I don't have to have contact with NAV.*

- *Og kanskje skaffe meg noen jobber selv etter hvert, og bygge det opp. En fordel med uføretrygd er jo at jeg slipper å ha kontakt med NAV.*

Q2.72 *But when she said that you can go back to work again, it was in the old days that when you became disabled, you were done. But when I finally sort of understood that it would calm me down so much. Then everything with the reporting cards and all that would become easier because you don't have to deal with that. And that was absolutely true*

- *Men da hun fikk sagt at du kan gå ut i jobb igjen, det var de gamle dager det at du liksom ble ufør, så var du ferdig. Men så når jeg endelig liksom forstod at det ville roe det så mye ned for meg. Da ville det liksom bli lettere med alt dette med meldekort og alt at du slipper å drive med det. Og det var helt sant det.*

Q2.73 Because I thought that maybe if I just get disability benefits now, I could sort of settle down a bit, so that I can start to focus on, not NAV at least, but the rest of my life, in order to try to get better.

- for jeg tenkte kanskje sånn at hvis jeg bare blir ufør nå, så kanskje jeg på en måte får landet litt, sånn at jeg kan begynne å fokusere på, ikke NAV i hvert fall, men da resten av min, for å prøve å bli frisk da.

Q2.74 What makes me think… that when I apply for disability benefits, I will probably breathe a big sigh of relief, because then I am done and I can focus more on maybe partially returning to some form of working life in the future

- det som gjør at når jeg søker ufør så tenker jeg da kommer jeg nok til å puste veldig lettet ut, for da er jeg ferdig og jeg kan fokusere mer på å kanskje komme delvis tilbake i en eller annen form for arbeidsliv i fremtiden.

Q2.75 So now that I have become disabled, I manage to make dinner for the kids every day, I do the laundry, I keep things in order, but then I don't have to think about anything else. I don't have to be somewhere else, because I have thought that maybe I can work a few hours. I have noticed now that I have become disabled, that I have that little extra energy to help others. And I feel that it gives me a lot. And I feel that they give me a lot by being able to help others. And now I have the energy for it. I have never had the energy to be able to do something like that before.

- Så som nå som jeg har blitt ufør, så klarer jeg å lage middag til barna hver dag, jeg vasker klær, jeg holder orden, men da slipper jeg å tenke på noe annet. Jeg skal ikke være noe annet sted, for jeg har jo tenkt at kanskje jeg klarer å jobbe noen timer Jeg har merket nå etter at jeg har blitt ufør, at jeg har den litt ekstra energien til å hjelpe andre. Og det synes jeg gir meg veldig mye. Og føler at de gir meg veldig mye å kunne hjelpe andre. Og nå har jeg energi til det. Jeg har aldri hatt energi til å kunne gjøre noe sånt før.
